# Purification and biological activity of natural variants synthesized by tridecaptin M gene cluster and *in vitro* drug-kinetics of this antibiotic class

**DOI:** 10.1038/s41598-019-54716-8

**Published:** 2019-12-11

**Authors:** Manoj Jangra, Manpreet Kaur, Mansi Podia, Rushikesh Tambat, Vidhu Singh, Nishtha Chandal, Nisha Mahey, Navdezda Maurya, Hemraj Nandanwar

**Affiliations:** Clinical Microbiology & Bioactive Screening Laboratory, CSIR-Institute of Microbial Technology, Sector -39A, Chandigarh, 160036 India

**Keywords:** Antibiotics, Drug discovery

## Abstract

The flexibility of the adenylation domains of non-ribosomal peptide synthetases (NRPSs) to different substrates creates a diversity of structurally similar peptides. In the present study, we investigated the antimicrobial activity of different natural variants synthesized by tridecaptin M gene cluster and performed the *in vitro* drug kinetics on this class. The natural variants were isolated and characterized using MALDI-MS and tandem mass spectrometry. All the peptides were studied for their antimicrobial activity in different pathogens, including colistin-resistant bacteria, and for haemolytic activity. Furthermore, *in vitro* drug kinetics was performed with tridecaptin M (or M_1_, the major product of the gene cluster). The natural variants displayed a varying degree of bioactivity with M_11_ showing the most potent antibacterial activity (MIC, 1–8 µg/ml), even against *A. baumannii* and *P. aeruginosa* strains. The *in vitro* kinetic studies revealed that tridecaptin M at a concentration of 16 µg/ml eradicated the bacteria completely in high-density culture. The compound demonstrated desirable post-antibiotic effect after two-hour exposure at MIC concentration. We also observed the reversal of resistance to this class of antibiotics in the presence of carbonyl cyanide *m*-chlorophenyl hydrazine (CCCP). Altogether, the study demonstrated that tridecaptins are an excellent drug candidate against drug-resistant Gram-negative bacteria. Future studies are required to design a superior tridecaptin by investigating the interactions of different natural variants with the target.

## Introduction

The evolution of antibiotic resistance is a stochastic process that magnifies under the selection pressure of drugs^1^. Antimicrobial resistance exacerbates morbidity and mortality. Additionally, it impacts the economic status of a nation’s health care systems, because of the prolonged treatments in ICUs^2–4^. For instance, in Europe, the antibiotic-resistant infections cost around $1.5 billion per annum, while in the U.S., the estimated cost is $55 billion^5^. Due to the novel resistance mechanisms emerging, antimicrobial resistance has become a health crisis, making common infections complicated and untreatable.

Among the priority pathogens addressed by the World Health Organization (WHO) and the Centers for Disease Control and Prevention (CDC), Gram-negative bacilli (GNB) are most refractory to many antibiotics used in clinical settings^5,6^. Urgent therapies are required to bridge the gap between the emerging resistance in these superbugs and the lack of new antibiotics. In the last few years, several reports have published on an old class of antibiotics, i.e., tridecaptin, which selectively targets Gram-negative bacteria^7–10^. Recently, tridecaptin M has been shown to demonstrate its antibacterial effects in colistin-resistant *Enterobacteriaceae*^11^. Tridecaptin M showed superior activity in a murine thigh infection model of colistin-resistant *Klebsiella pneumoniae* strain, compared to colistin. These results make this class an attractive drug candidate, which should be improved to enhance the efficacy or to minimize the toxicity. The efforts to build a structure-activity relationship on tridecaptin A_1_ indicated the importance of D-Trp5, D-Dab8 and D-*allo*-Ile12 in the antimicrobial activity^12^. Attempts have been made, in the past, to design the superior tridecaptins^10,13^. Unfortunately, most of the tridecaptin analogues either exhibited similar activity as the natural variant or found to be less potent. One ot two analogues possessed a better antimicrobial activity against specific pathogens. Nonetheless, no chemical variant has shown significant reduction in efficacy against different species of bacteria.

Natural variants are also described for tridecaptin A, tridecaptin B and tridecaptin C^8,14,15^. These variants differ from each other, either at the fatty acid part or at one or more amino acid positions. The comparative analysis of their biological activities or detailed drug kinetics of this class, however, have not been investigated. There is a lack of information whether one variant is superior to another. During the isolation of tridecaptin M from a mud bacterium, *Paenibacillus* sp. M-152, we observed several peaks with activity against *K. pneumoniae*. Upon chemical characterization, we confirmed that they all belong to tridecaptin M family, and are the products of a single biosynthetic gene cluster, present in the whole genome of M-152 strain. The present study describes the different natural variants of this cluster, and their comparative biological activity against different pathogens, including colistin-resistant *Escherichia coli* and *K. pneumoniae* strains. Moreover, the variants were studied for their haemolytic activity. Finally, we performed the *in vitro* drug kinetics on this class, in order to develop a future drug candidate against Gram-negative pathogens.

## Methods

### Purification of different natural variants of tridecaptin M by reversed-phase high-pressure liquid chromatography (RP-HPLC)

**Figure 1:**
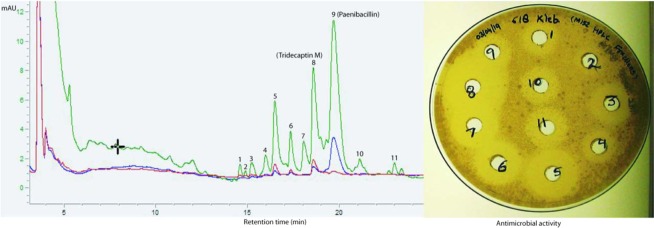
RP-HPLC chromatogram of cation-active fraction, showing the different peaks active against *K. pneumoniae*. In the right panel, the antimicrobial activity of different peaks is depicted. In the HPLC chromatogram, green colour indicates the absorbance at 220 nm, blue 254 nm and red 280 nm.

The strain M-152 was inoculated in 700 ml MHB in 2 L Erlenmeyer flask, and the crude fermentation extract preparation, ion-exchange chromatography and the first step of RP-HPLC were performed as described for tridecaptin M^11^. The peaks obtained in HPLC were assayed against *K. pneumoniae* using agar well diffusion method. The active peaks (Fig. 1) were purified by the second round of HPLC under different conditions, optimized for each compound. The mobile phase system for all the peaks consisted of solvent A as 20% acetonitrile in Milli-Q and solvent B as 100% acetonitrile. Both solvents contained 0.075% TFA. C_18_ column (Waters, XBridge, 10 mm × 250 mm, 5 µm, 130 Å) was used to purify the compounds. Following gradient was used for the resolution of different components; for P1, 8% B to 26% B in 30 min, for P3, 8% B to 25% B in 23 min, for P4, 8% B to 23% B in 22 min, for P5, 3% B to 21.5% B in 24 min, for P6, 10% B to 25% B in 25 min, for P7, 8% B to 28% B in 25 min, for P8, 8% B to 26% B in 23 min, for P10, 10% B to 28% B in 30 min, for P11, 10% B to 28% B in 31 min. In two to three min, the gradient was reversed to initial column conditions and was equilibrated for five to six min, before the next injection. Multiple injections were given to generate a sufficient quantity of each compound for further studies.

### Mass spectrometry

All the purified compounds were subjected to MALDI-MS and MALDI-MS/MS analysis. The daughter ions generated upon fragmentation were assigned manually and the spectra were compared with MS/MS pattern of tridecaptin M^11^.

### Minimum inhibitory concentration (MIC) determination and haemolysis assay

MICs for all the compounds were determined by broth microdilution assay, according to CLSI guidelines^16^ as described previously^11^. Haemolysis experiments were performed with fresh rabbit RBCs^11^. All the compounds were tested at a concentration of 128 µg/ml. To obtain rabbit blood, the experiments and the protocols were in accordance with the ethical standards of the Institutional Animal Ethics Committee (IAEC) of the Institute of Microbial Technology approved (Approval no. IAEC/17/11) by Committee for the Purpose of Control And Supervision of Experiments on Animals (CPCSEA, Government of India).

### Effect of CFU concentration on MIC

*K. pneumoniae* ATCC 700603 cells were grown to an OD_600_ of 3.0 (>10^9^ CFU/ml) and diluted in fresh CA-MHB to obtain five different cell suspensions ranging from 10^5^ to 10^9^ CFU/ml. The MIC of tridecaptin M was determined for each condition using 3-(4,5-dimethylthiazol-2-yl)-2,5-diphenyltetrazolium bromide (MTT). The bacteria were incubated with the compound, at concentrations ranging from 64 µg/ml to 0 µg/ml in a 2-fold manner, in a 96-well plate. Eighteen hours after the incubation, 20 µL of MTT (5 mg/ml) was added to all the wells, and the plate was incubated for another one hour. The colour change in wells was observed and the MICs were calculated as the concentration at which no colour change in the well was observed.

### Time-kill studies

*K. pneumoniae* ATCC 700603 was grown in CA-MHB and OD_600_ was adjusted to 0.15 (~10^8^ CFU/ml). One-eighty microliters of this culture were added to 96-well plate and treated with different concentration of tridecaptin M (20 µL). The plate was incubated in Biotek reader at 37 °C, with shaking and OD_600_ was measured at the 30-min interval. In control wells, 20 µL of PBS was added. Time-kill kinetics was also performed using the spread-plating method. Briefly, 10^8^ CFU/ml of cells were incubated with 2 × MIC (i.e., 16 µg/ml). At different time points, the sample was taken and spread plated on MHA plates after appropriate dilution. The colonies were counted and expressed as CFU/ml. The experiments were performed in triplicate and repeated at least three times.

### Post-antibiotic effect

*K. pneumoniae* cells (~10^8^ CFU/ml) were treated with different concentrations of tridecaptin M in one millilitre of CA-MHB in microcentrifuge tubes. After short exposure, i.e., 0.5 h, 1 h and 2 h, the sample was centrifuged and resuspended in PBS. Twenty microliters of the sample were added in 180 µL of fresh CA-MHB and growth kinetics was studied, with incubation at 37 °C and medium shaking, in Biotek reader by the measurement of OD_600_ for six hours at a 30-min interval. The cells without peptide were taken as positive control and were processed under similar conditions. The readings were measured in triplicate, with two biological repeats.

### *In vitro* drug kinetics

*K. pneumoniae* ATCC 700603 cells (10^9^ CFU/ml) were incubated with tridecaptin M at a concentration of 1 × MIC (16 µg/ml) in one millilitre CA-MHB. The cells were also suspended in CA-MHB supplemented with fetal bovine serum (FBS) at a final concentration of 5% (vol/vol). At different time points until 24 h, one tube was taken and centrifuged at 12,000 *g* to remove the biomass. The cell-free supernatant was assessed for measurement of the remaining unbound peptide using RP-HPLC, and the concentration was calculated by the standard graph of known concentrations of peptide vs. corresponding peak area. Five per cent FBS solution in CA-MHB was also incubated with peptide alone to check the stability over time.

### Synergy assays

*Acinetobacter baumannii* ATCC 19606, *Pseudomonas aeruginosa* ATCC 27853, *K. pneumoniae* ATCC 700603, *K. pneumoniae* P3R, *K. pneumoniae* GMCH 13 and *K. pneumoniae* GMCH 15 were grown in CA-MHB and adjusted to ~4 × 10^5^ CFU/ml. 100 µl of fresh CA-MHB containing carbonyl cyanide *m*-chlorophenyl hydrazine (CCCP) or phenylalanine-arginine beta-naphthylamide (PA*β*N) (at a final concentration of their 1/4^th^ of MIC or 1/8^th^ of MIC) was added to the wells of a 96-well flat bottom plate. Tridecaptin M was diluted in a 2-fold manner. 100 µl of culture was added and the plates were incubated for 16 to 18 h at 37 °C and observed for MICs. The fold modulation in MICs of tridecaptin M was calculated by comparing the results in the presence or absence of CCCP and PA*β*N.

### Nile red efflux inhibition assay

To assess the effect of CCCP on efflux in *A. baumannii* ATCC 19606, the Nile Red efflux assay was used^17^. The overnight grown culture was centrifuged at 3000 *g* for 10 min at room temperature. The pellet was resuspended in 20 mM potassium phosphate buffer (pH 7.0) containing 1 mM MgCl_2_ and washed twice with the same buffer. The cells were adjusted to an OD_660_ of 1.0 and the aliquots were transferred to a glass tubes containing CCCP at a final concentration of 4 mg/L. After 15 min, Nile Red was added to a final concentration of 5 μM, and the cell suspension was incubated in a shaker (140 rpm, 37 °C) for 3 h. The cells were kept at room temperature for 60 min and centrifuged for 5 min at 3000 *g*. The supernatant was discarded and the cell pellet was resuspended in assay buffer. Next, Nile Red efflux was immediately triggered by rapid energization with 50 mM glucose and the fluorescence was recorded at excitation and emission wavelength 552 nm and 636 nm, respectively, over a period of 5 min.

### Membrane permeabilization and depolarization assay

For outer membrane permeabilization study, NPN dye was used as described previously^11^. *A. baumannii* ATCC 19606, *P. aeruginosa* ATCC 27853, *K. pneumoniae* ATCC 700603 were treated with two different concentrations of the peptide (4 µg/ml and 8 µg/ml). To check alteration in the cytoplasmic membrane potential, DiSC_3_(5) probe was used^11^.

## Results

### Isolation and chemical characterization of different peptide variants

The cation-active fraction of crude extract, prepared from the fermentation of M-152 strain, displayed several peaks in RP-HPLC chromatogram. The peaks, spanning the region of retention time (RT) of 14 min to 24 min, showed antimicrobial activity against *K. pneumoniae* (Fig. 1). Each peak was collected in a separate fraction and was successfully purified, except fraction-4 which contained two fused peaks. These peaks could not be separated in HPLC and were studied as a mixture. The fraction-2 did not show bioactivity. Moreover, it was produced in very low yield, and therefore, not processed further. A total of eleven variants were successfully resolved and purified (Supplementary Figs S1–S10), to identify and study their biological activity. The peptides were characterized by tandem mass spectrometry and their mass spectra were compared with that of tridecaptin M to locate the amino acid residues’ positions where the variants showed structural differences. In MS/MS spcectra, there were some prominent peaks present which did not belong to any b- or y-ion and therefore these peaks are not assigned in the spectra. All the b- and y-ions present were assigned manually and their de novo sequences were determined (Supplementary Figs S11 to S21; Table 1). Out of 11 variants, six were characterized completely. Mass spectrometry results revealed that the lipid tail was consistent among all the variants (for those which were characterized successfully) because the peptides had differences only in their amino acid part which was well satisfied with their MS/MS spectra. Moreover, in all the natural tridecaptins reported till date, only *S*-configuration of methyl group is present (because they are derived from L-isoleucine)^8,18^. Also, the chirality does not contribute significantly in the activity. Therefore, we did not take into account the composition of lipid part and the lipid moiety at N-terminus was considered 6(*S*)-methyloctanoic acid in all the characterized peptides. The originally described variant and the major product of this cluster, i.e., tridecaptin M^11^ was considered M_1_, and the other variants were named according to their order of elution (and therefore hydrophobicity) in HPLC. The compound M_2_ eluted in the beginning and was most hydrophilic among all. In its amino acid sequence, both the isoleucine residues, present at position 11 and 12, were replaced by less hydrophobic residue, valine. Similarly, the last variant, M_11_ had isoleucine (at position nine) replaced by phenylalanine, and therefore, possessed greater hydrophobicity. In M_5_ and M_8_, the tryptophan5 is replaced by phenylalanine, indicating the reduction in absorbance at 280 nm. This difference was clearly visible in HPLC chromatogram (Fig. 1), where these two peaks showed a significant decrease in absorbance intensity at 280 nm, compared to other variants. Table 1 describes the molecular masses and the amino acid sequences of different variants. We could not obtain the MS/MS spectra of M_3_, M_4_, M_9_, and M_10_ and therefore, these variants are not characterized yet. M_11_ was partially characterized using MS/MS spectrum. The MS data of these uncharacterized variants are given in Supplementary Table S1. The unavailability of compounds due to the extremely low yield limited the characterization at this stage. The method development to enhance the yield in fermentation is currently under process.

**Table 1:** Amino acid sequences of natural variants of tridecaptin M


Peptide	MW	Amino acid sequence												
		1	2	3	4	5	6	7	8	9	10	11	12	13
M_1_ (or M)	1487.8	Gly	D-Dab	Gly	D-Ser	D-Trp	Ser	Dab	D-Dab	Ile	Glu	Ile	D-*a*Ile	Ser
M_2_	1459.8	Gly	D-Dab	Gly	D-Ser	D-Trp	Ser	Dab	D-Dab	Ile	Glu	Val	D-Val	Ser
M_5_	1434.8	Gly	D-Dab	Gly	D-Ser	D-Phe	Ser	Dab	D-Dab	Ile	Glu	Ile	D-Val	Ser
M_6_	1473.8	Gly	D-Dab	Gly	D-Ser	D-Trp	Ser	Dab	D-Dab	Ile	Glu	Ile	D-Val	Ser
M_7_	1473.8	Gly	D-Dab	Gly	D-Ser	D-Trp	Ser	Dab	D-Dab	Val	Glu	Ile	D-*a*Ile	Ser
M_8_	1448.8	Gly	D-Dab	Gly	D-Ser	D-Phe	Ser	Dab	D-Dab	Ile	Glu	Ile	D-*a*Ile	Ser
M_11_	1568.8	ND	ND	ND	ND	ND	ND	ND	D-Dab	Phe	Glu	Ile	D-*a*Ile	Ser

ND= not determined due to unavailability of the compound. The highlighted residues show the amino acid positions where the natural variants differ.

### Antimicrobial profile and haemolytic activity

**Figure 2:**
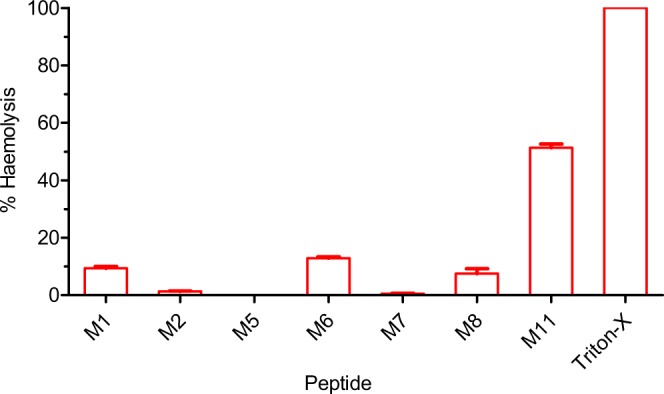
Haemolytic activity of different variants. All the peptides were tested at a concentration of 128 µg/ml. The data represent mean ± SD values of three replicates.

The natural variants of tridecaptin M exhibited significant differences in their MIC values against different pathogens (Table 2). The peptide M_11_ had the lowest MICs against most of the strains tested, compared to the other variants. This variant showed a MIC value of 1 µg/ml in the colistin-resistant clinical isolates of *K. pneumoniae*. Notably, the peptides were also active against *Proteus mirabilis* and *Serratia marcescens* strains, which are intrinsically resistant to polymyxins^19^. The peptide M_1_ or the other variants showed higher MICs in *A. baumannii* or *P. aeruginosa* strains. These results were consistent with the previously reported activity of tridecaptins against these organisms^7,8,20^. The resistant strain P3R was generated by the sequential passaging of *K. pneumoniae* ATCC 700603 in the sublethal concentrations of tridecaptin M^11^. Most of the variants showed cross-resistance in this strain, indicating that these peptides share a common structural scaffold which is responsible for the activity and hence have a similar resistance mechanism. However, the peptides M_5_, M_6_,M_7_ and M_11_ displayed significantly lower MIC values in this strain as compared to M_1_. The peptide M_11_ also exhibited excellent activity in *A. baumannii* and *P. aeruginosa* strains, with a MIC of 4 µg/ml and 8 µg/ml respectively. The haemolytic tendency of the peptides also varied significantly (Fig. 2). The compound M_11_ displayed the highest degree of haemolysis (~50%), whereas M_2_ M_5_, and M_7_ were less toxic than the other variants.

**Table 2:** Antimicrobial activity profile of different variants against indicator strains.

Bacterial strain	Peptide MIC (µg/ml)						
	M (orM_1_)	M_2_	M_5_	M_6_	M_7_	M_8_	M_11_
*K. pneumoniae* ATCC 700603	4	8	16	4	8	4	2
*K. pneumoniae* AH-3 (Col-R)	4	8	16	4	8	8	1
*K. pneumoniae* AH-16 (Col-R)	4	>16	16	2	8	4	1
*E. coli* CF-23 (*mcr-1*)	4	16	16	4	16	4	1
*Proteus mirabilis* MTCC 1429	>16	>16	16	>16	>16	>16	4
*Serratia marcescens* MTCC 97	8	>16	8	16	8	16	4
*K. pneumoniae P3R* (M_1_-R)	64	>32	16	16	16	128	8
*K. pneumoniae* GMCH 13	8	ND	ND	ND	ND	ND	4
*K. pneumoniae* GMCH 15	16	ND	ND	ND	ND	ND	2
*A. baumannii* ATCC 19606	64	>32	>32	>32	>32	128	4
*P. aeruginosa* ATCC 27853	>128	>32	>32	>32	>32	>128	8

ND= not determined.

### *In vitro* drug kinetic studies

**Figure 3:**
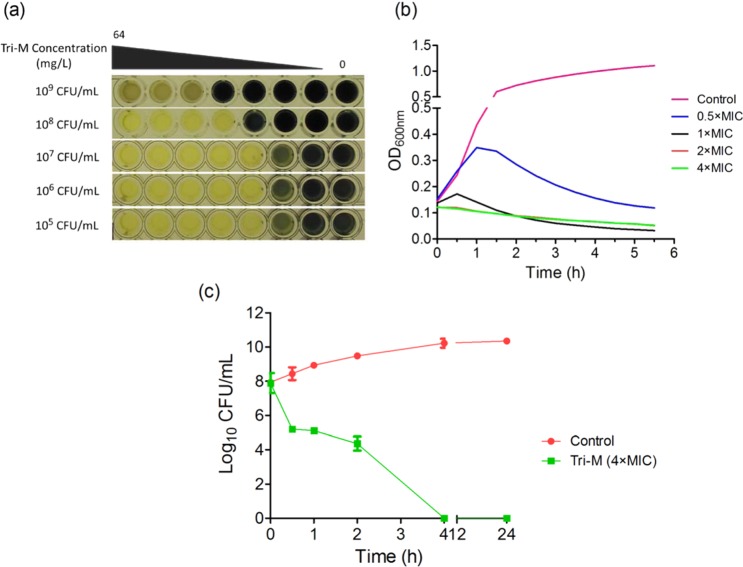
Effect of bacterial load on the MIC of tridecaptin M (or M_1_) and time-kill kinetics. (**a**) MICs of tridecaptin M at various CFU/ml concentrations of *K. pneumoniae* ATCC 700603. The results are expressed as mode MIC values obtained from four independent experiments. Tri-M, tridecaptin M_1_. (**b**) Time-kill assay using optical density (OD) measurements at 600 nm in the presence of different concentrations of tridecaptin M. Control represents the untreated sample. The experiment was performed in triplicate, with two biological repeats. (**c**) Time-kill studies using spread plating method. Similar results were obtained as depicted in (**b**). Tridecaptin M_1_ caused complete eradication of bacteria in 4 hours.

**Figure 4:**
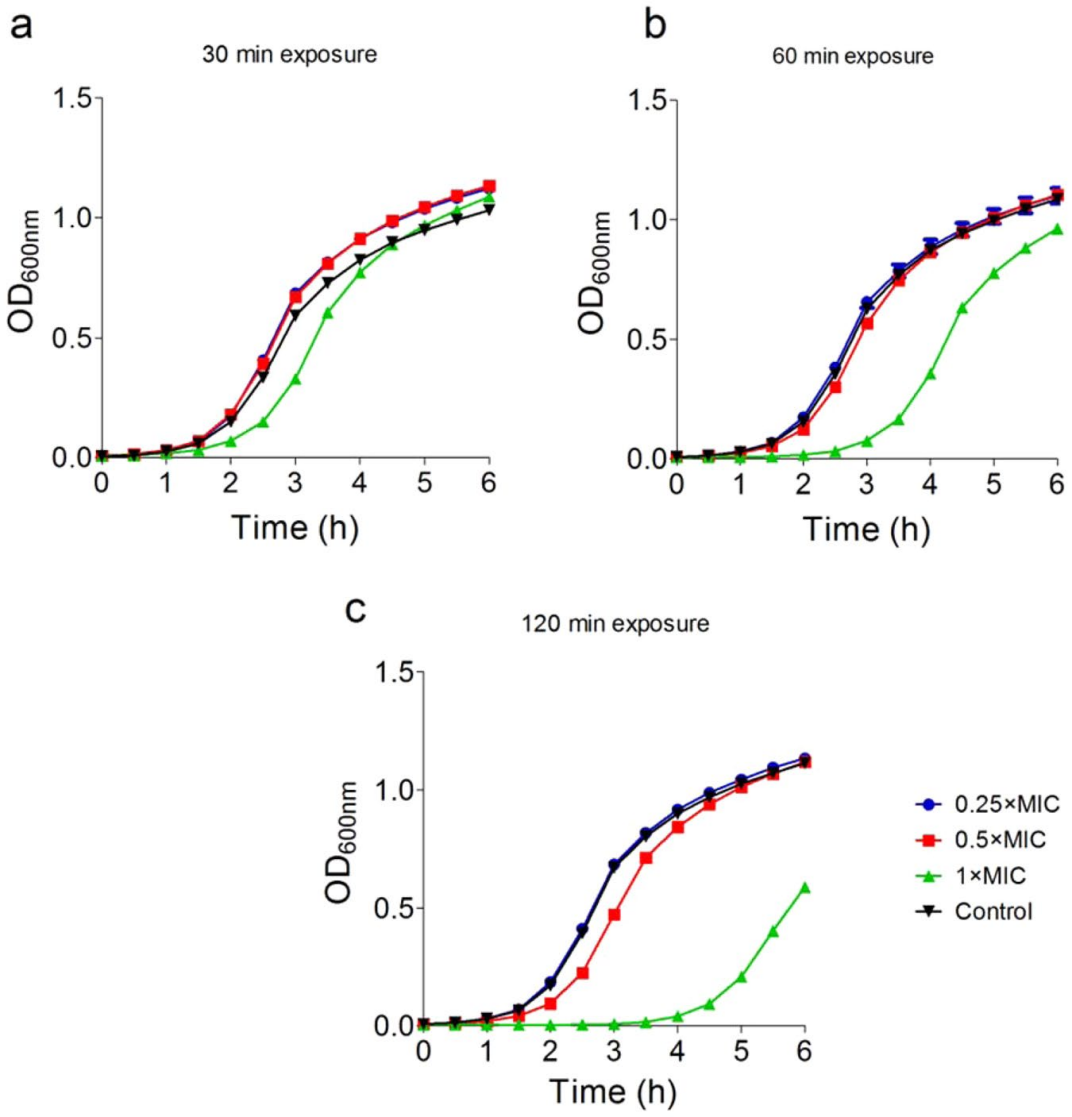
Post antibiotic effect of tridecaptin M after (**a**) 30 min (**b**) 60 min and (**c**) 2 h of exposure. The bacteria showed suppressed survival for approximately four hours, after 2 h exposure at a concentration of 1 × MIC. The experiments are performed in triplicate, with two biological repeats.

Owing to extremely-low yields of the variants, we conducted drug kinetic studies with tridecaptin M (or M_1_). As visible in Fig. 3a, the increasing concentrations of CFU were not directly proportional to the MIC values of the peptide. Even at the 100-fold increase in CFU, the MIC value was consistent at 4 µg/ml. Surprisingly, a CFU concentration of >10^9^ CFU/ml was completely inhibited by the compound at 16 µg/ml. Since the high density of culture interferes with the optical density measurements, the time-kill kinetics was performed with a lower concentration of bacteria. The peptide, at a concentration of its MIC value or above, resulted in the reduction of optical density (Fig. 3b), indicating the lysis of bacteria. The similar results were obtained in spread plating method (Fig. 3c), and the bacteria were killed completely in four hours. Based on these data, we designed the experiments to study the post-antibiotic effect (PAE) of tridecaptin M, after a short exposure. Figure 4 depicts the survival suppression of bacterial growth after the short-time exposure to tridecaptin M at different concentrations. At subinhibitory concentrations, no PAE was observed. In fact, the bacterial growth favoured after 30 min of treatment at sublethal concentrations. Similar results were obtained in time-kill studies (Fig. 3b) where the optical density increased for an initial one hour and then started to decline. A two-hour exposure at 1 × MIC concentration halted the bacterial growth for around four hours.

**Figure 5:**
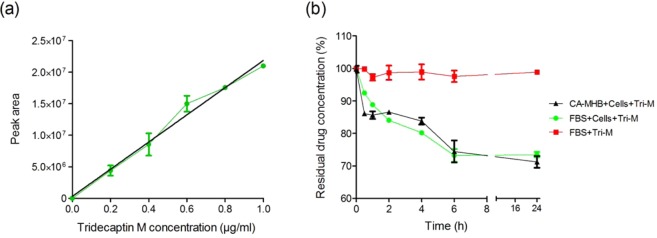
*In vitro* drug kinetics of tridecaptin M (**a**) Standard calibration graph of concentration vs. peak area, determined by RP-HPLC. The data plotted are Mean ± SD of three replicates. (**b**) Concentration of free or unbound peptide over time when incubated with *K. pneumoniae in vitro* in cation-adjusted MHB and foetal bovine serum (5% vol/vol). The peptide was found to be stable in the presence of serum. In six hours, 25% to 30% of the compound is utilized, indicating that the remaining peptide is present in the free or unbound form in the solution. At six hours, the bacteria were killed completely, as observed in time-kill assay (Fig. 2(c)). The experiment was performed in triplicate and repeated two times.

The measurement of the unbound free drug in the body fluids can be correlated with the dosage regimen and can help design the dosage strategies. We sought to determine the unbound peptide concentration under *in vitro *conditions using RP-HPLC. A standard graph of known concentration vs. corresponding peak area was plotted to calculate the concentration of peptide in the sample (Fig. 5a). The peptide acted slowly on bacteria, with 75% residual concentration left in six hours, and there was no further significant decrease in the concentration at 24 h (Fig. 5b). The presence of FBS did not affect the stability of the compound. The peptide was used at 1 × MIC concentration (16 µg/ml) with >10^9^ CFU/ml of bacteria. These results indicate that only a fraction of the compound is required to exert the bactericidal effect, even in very high-density culture.

### Synergy studies

Tridecaptin M or the other variants (except M_11_) displayed higher MICs in *A. baumannii* and *P. aeruginosa* strains. To investigate in detail why this class had narrow spectrum selectivity, the susceptibility of tridecaptin M in these strains was checked in the presence of two efflux pump inhibitors (EPIs). *K. pneumoniae* GMCH 13 and *K. pneumoniae* GMCH 15 were studied, because these strains were less susceptible to tridecaptin M, compared to other *K. pneumoniae* isolates. In *A. baumannii* strain, CCCP reduced the MIC of tridecaptin M by 16-fold, from 64 µg/ml to 4 µg/ml, indicating the complete reversal of resistance in this strain (Table 3). Notably, CCCP inhibited the efflux of Nile red dye (a substrate for efflux pumps) in *A. baumannii strain*, suggesting the presence of efflux pumps (Supplementary Fig. S22). In contrast, PA*β*N did not show any synergy. Moreover, no synergy was observed in *P. aeruginosa*, P3R and *K. pneumoniae* ATCC 700603 strains with either of the efflux pump inhibitors. In GMCH 13 and GMCH 15 strains, the MICs of the peptide were reduced by two-and four-fold respectively, indicating the role of efflux pumps in resistance. We also observed a four-fold modulation in the activity of M_11_ against *A. baumannii* strain in the presence of CCCP (Supplementary Table S2). The probable reason for low synergistic effects obtained for M_11_ is that M_11_ had lower intrinsic MIC in this strain, compared to M_1_.

**Table 3:** Synergistic activity of tridecaptin M in the presence of efflux pump inhibitors.

Strain	Intrinsic Activity; MIC (*µ*g/ml)			MIC (*µ*g/ml) of Tri-M in the presence of EPIs				FICI	
				PA*β*N		CCCP		CCCP	
	Tri-M	PA*β*N	CCCP	1/4^th^ MIC	1/8^th^ MIC	1/4^th^ MIC	1/8^th^ MIC	1/4^th^ MIC	1/8^th^ MIC
*A. baumannii* ATCC 19606	64	64	64	64	64	4	8	0.31	0.25
*P. aeruginosa* ATCC 27853	256	64	64	256	256	256	256	—	—
*K. pneumoniae* ATCC 700603	2	256	128	2	2	2	2	—	—
*K. pneumoniae* P3R (Tri-M Resistant)	64	256	128	64	64	64	64	—	—
*K. pneumoniae* GMCH 13	8	256	32	8	8	4	8	0.75	—
*K. pneumoniae* GMCH 15	16	256	64	16	16	4	8	0.5	0.62

Tri-M= Tridecaptin M (or M_1_).

### Membrane permeabilization and depolarization assays

**Figure 6:**
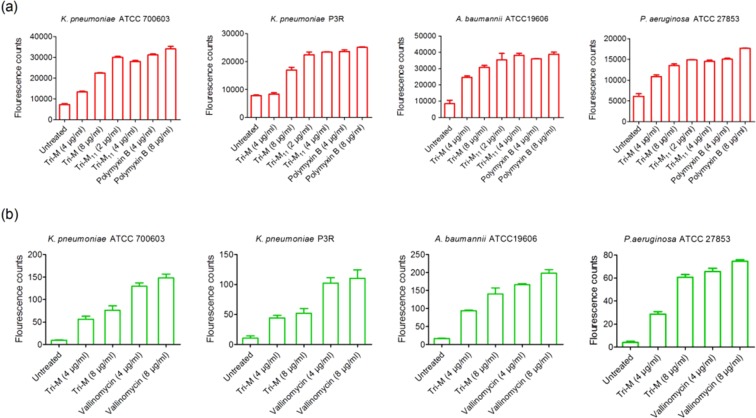
Effect of tridecaptin M on the outer and cytoplasmic membranes of various pathogens. (**a**) NPN assay to determine the disruption of outer membrane. M_1_ showed positive results in all the strains, despite of its high MIC in P3R strain, *A. baumannii* and *P. aeruginosa*. M_11_ demonstrated greater uptake of NPN, compared to M_1_, even a lower concentration. (**b**) Membrane depolarization assay with DiSC3(5). Similar results were obtained for M_1_, as observed in the NPN assay. This data suggests that the resistance to tridecaptin M in these strains (excluding ATCC 700603) may be due to efflux pumps or some other mechanisms. Both the experiments were conducted in triplicate, with three biological repeats.

As reported previously, tridecaptin A_1_ interacts with lipopolysaccharide to permeabilize the outer membrane, before its binding to lipid II^9^. To study the effects of tridecaptin M on the outer and the inner membranes of bacteria, either sensitive or resistant to this peptide, we initially selected the NPN dye. As expected, the peptide showed the NPN uptake in *K. pneumoniae* ATCC 700603 (Fig. 6a). Surprisingly, we could also observe NPN uptake in *A. baumannii* strain in the presence of M_1_, despite its high MIC against this bacterium. These results clearly demonstrate the interaction of this compound with the outer membrane. The positive results in P3R strain with M_1_ referred to the involvement of different resistance mechanisms, not an alteration in the outer membrane. Similar results were obtained in *P. aeruginosa* strain, suggesting that the tridecaptin binding sites in the outer membranes of Gram-negative bacteria are structurally similar. Of note, M_11_ showed greater NPN uptake, even at a lower concentration, and the results were comparable to those obtained for polymyxin B. In membrane depolarization assay, M_1_ caused an increase in fluorescence of DiSC_3_(5) dye in all the strains, indicating an alteration in the membrane potential (Fig. 6b). These results confirmed that tridecaptin M can penetrate the outer membrane of *A. baumannii* or *P. aeruginosa* at a similar concentration as used for *K. pneumoniae* and can disturb the proton motive force. Yet it remained unclear why these strains showed a higher MIC to this peptide. M_11_ interfered with the fluorescence of DiSC_3_ dye because we observed the negative results at a higher concentration in all the organisms tested (data not shown).

## Discussion

Several non-ribosomal peptide (NRP) antibiotics, e.g., polymyxins, vancomycin, daptomycin, bacitracin are used in clinical settings^21^. This is noteworthy that most of these peptides are present as a mixture of several closely related components, encoded by a single biosynthetic gene cluster. This diversity is incorporated by the flexible substrate specificities of some of the adenylation modules. Tridecaptins also belong to NRP class and are synthesized in fermentation as a mixture of different variants. Tridecaptin M differs from tridecaptin A and tridecaptin C at positin 1, 2 and in their lipid composition^14^. Also, tridecaptin A has alanine at position 13 while tridecaptin M has a serine. Tridecaptin M showed close similarity with tridecaptin B; however, the major difference lies at the position 11 where tridecaptin B has a valine but the tridecaptin M has an isoleucine. Whether these amino acid substitutions at some places in the peptide chain affect the antimicrobial activity is poorly understood. We could observe some discrepancies in the substrate preferences of adenylation domains of tridecaptin M gene cluster^11^, consistent with the previous reports^8,20^. We were interested to study the effects of these substrate specificities in adenylation modules, resulting in the production of the natural diversity of compounds, on the antimicrobial activity. A total of 11 variants were isolated, which showed dissimilar bioactivity against various pathogens. The Ile9Val replacement reduced the MIC by two- to four-fold, as observed in M_1_ and M_7_. The position 12 was either occupied by D-aIle or D-val. This replacement had no effect on the MIC of M_1_ and M_6_ variants. This was surprising because, in tridecaptin A_2_ variant, the valine caused two- to four-fold reduction in the MIC^22^. Presumably, this might be due to the synergistic effects of amino acid residue at this position with the other residues in the peptide chain. For instance, the replacement of both Trp5 and D-aIle12 in M_5_ by Phe and D-val respectively caused a four-fold decrease in the activity, whereas none of the single replacement alone showed any effect. Likewise, M_2_ that contained valine residues at both 11 and 12 positions had two- to eight-fold less activity. The amino acid sequences of tridecaptin A variants differ from that of tridecaptin M_1_ at four positions and also, they vary in their fatty acid composition^14^. Thus, it can be concluded that the role of a particular amino acid is specific to the different peptides of this class, and that the similar replacements in these peptides at specific position may not have a similar effect on their activity. Despite their structural similarities, some variants such as M_5_, M_6_ M_7_ and M_11_ showed lower MIC values in P3R strain (M_1_-resistant mutant). These results suggested that their mechanism of interaction with their target or their binding affinities with the target may be different from each other. The peptides also exhibited varying haemolytic activity, with M_11_ being the most haemolytic in nature.

We further studied the *in vitro* drug kinetics of this class of antibiotics to examine how this can be correlated with the *in-vivo* systems. Tridecaptin M showed just two- to four-fold increase in the MIC while increasing the bacterial load by 1,000 to 10,000-fold. One plausible explanation for this phenomenon is that the antibiotics entry in the bacteria or on membrane or cell wall depends on diffusion. Not the entire compound is used in killing of the cells. As visible in *in vitro* drug kinetics experiment that only 25–30% of the peptide was required to kill the bacteria completely, in high-density culture; remaining peptide was intact in the supernatant. Therefore, increasing the number of CFU may not show a liner relationship with antibiotic concentration. We proposed that a concentration (of free or unbound peptide) of 16 µg/ml or above in the blood would be sufficient to reduce the bacterial burden significantly in 24 h or less, even at a high CFU/ml.Tridecaptin M also depicted a reasonably good post-antibiotic effect (~4 hours) after a 2 h exposure at 1 × MIC concentration. Notably, the presence of efflux pump inhibitor, i.e. CCCP reversed the resistance of tridecaptin M in *A. baumannii* and *K. pneumoniae* strains. No synergy was obtained in the presence of PA*β*N; these results indicate that synergistic activity of CCCP with tridecaptins was either due to efflux pump ihbibition or some other mechanism such as membrane permeabilization or disruption of membrane potential. Further studies are required to investigate the exact mechiasm of synergy between these two compounds. Interestingly, we could observe the outer membrane permeabilization and alteration in membrane potential of *A. baumannii* or *P. aeruginosa* strains treated with M_1_ at a very low concentration (4 µg/ml or 8 µg/ml) which was equivalent to MICs in *K. pneumoniae* strains. These results suggest that this compound can successfully penetratein the these bacteria but some other factors are responsible for the resistance to this class of antibiotics.

To conclude, the study demonstrates that replacement of an amino acid residue by another counterpart at a specific position in the peptide chain has an effect on its antimicrobial activity. The variant M_11_ was the most potent among all the peptides, also showing excellent inhibition in *P. aeruginosa* and *A. baumannii* strains. The chemical characterization of M_9_, M_10_ and M_11_ is the future scope of this manuscript and the work is under process. There might also exist other tridecaptin clusters having adenylation domains with different substrate specifities that may lead to discovery of novel tridecaptins. Investigations are also required to study the interactions of different variants with their target, i.e., lipid II, in order to analyze the role of each amino acid in the binding and in the antimicrobial activity. These efforts, in future, may lead to designing of superior or next generation tridecaptins, to combat Gram-negative superbugs.

### Ethics

Rabbit blood was used for haemolysis assay. The experiments were conducted in compliance with the ethical standards of the Institutional Animal Ethics Committee (IAEC) of the Institute of Microbial Technology (Approval no. IAEC/17/11).

## Supplementary information

Supplementary information

## Data Availability

All the data related to this manuscript is available from the corresponding author upon request.
