# Supplementary material for: Purification and biological activity of natural variants synthesized by tridecaptin M gene cluster and in vitro drug-kinetics of this antibiotic class
Source: Sci Rep. 2019 Dec 11;9:18870. doi: 10.1038/s41598-019-54716-8 (PMC6906472; doi:10.1038/s41598-019-54716-8)
Supplement: Supplementary file 1 — Supplementary information [file 41598_2019_54716_MOESM1_ESM.pdf]

Supplementary information

Purification and biological activity of natural variants synthesized by tridecaptin M gene cluster and *in vitro* drug-kinetics of this antibiotic class

Manoj Jangra, Manpreet Kaur, Mansi Podia, Rushikesh Tambat, Vidhu Singh, Nishtha Chandal, Nisha Mahey, Navdezda Maurya, Hemraj Nandanwar*

Clinical Microbiology & Bioactive Screening Laboratory, CSIR-Institute of Microbial Technology, Sector -39A, Chandigarh, 160036, India

***Address for correspondence**

Dr Hemraj Nandanwar

Clinical Microbiology & Bioactive Screening Laboratory, CSIR - Institute of Microbial Technology, Sector -39A, Chandigarh, India, 160 036

E-mail: hemraj@imtech.res.in

Telephone: +91-172-6665338 Fax: +91-172-2690585/2690632

Supplementary Tables

Table S1 Mass spectrometric data of different variants of tridecaptin M

Peptide variant	Molecular formula $[M+H]^+$	Observed molecular weight $[M+H]^+$	Calculated monoisotopic mass $[M+H]^+$	Error (ppm)
M_1 (or M)	$C_{68}H_{114}N_{17}O_{20}$	1488.8246	1488.8426	18.0
M_2	$C_{66}H_{110}N_{17}O_{20}$	1460.8090	1460.8113	2.0
M_3	ND	1482.6736	ND	ND
M_4	ND	1460.6866	ND	ND
M_5	$C_{65}H_{111}N_{16}O_{20}$	1435.8076	1435.8160	8.0
M_6	$C_{67}H_{112}N_{17}O_{20}$	1474.7987	1474.8269	28.0
M_7	$C_{67}H_{112}N_{17}O_{20}$	1474.8167	1474.8269	10.0
M_8	$C_{66}H_{113}N_{16}O_{20}$	1449.7800	1449.8317	51.0
M_9	ND	1607.7673	ND	ND
M_{10}	ND	1563.8605	ND	ND
M_{11}	ND	1569.8368	ND	ND

Table S2 Synergistic activity of M_{11} with efflux pump inhibitor, CCCP

Strains	Intrinsic Activity; MIC (µg/ml)		MIC (µg/ml) of Tri-M_{11} in the presence of CCCP		FICI	
	Tri-M_{11}	CCCP	1/4th MIC	1/8th MIC	1/4th MIC	1/8th MIC
Acinetobacter baumannii ATCC 19606	4	64	1	2	0.5	0.625
Klebsiella pneumoniae GMCH 13	4	32	2	2	0.75	0.625
Klebsiella pneumoniae GMCH 15	2	64	2	2	-	-

Supplementary Figures

Sample Name	: M152-P3 actual 32ug/ml (XBridge C18 1 ml/min)
Sample ID	: 10-30%ACN/Water 0.075% TFA
Vail#	: 157
Injection Volume	: 50 uL
Data Filename	: Sample_133.lcd
Method Filename	: manoj.lcm
Batch Filename	:
Date Acquired	: 3/20/2019 8:38:29 PM
Data Processed	: 4/3/2019 3:11:25 PM

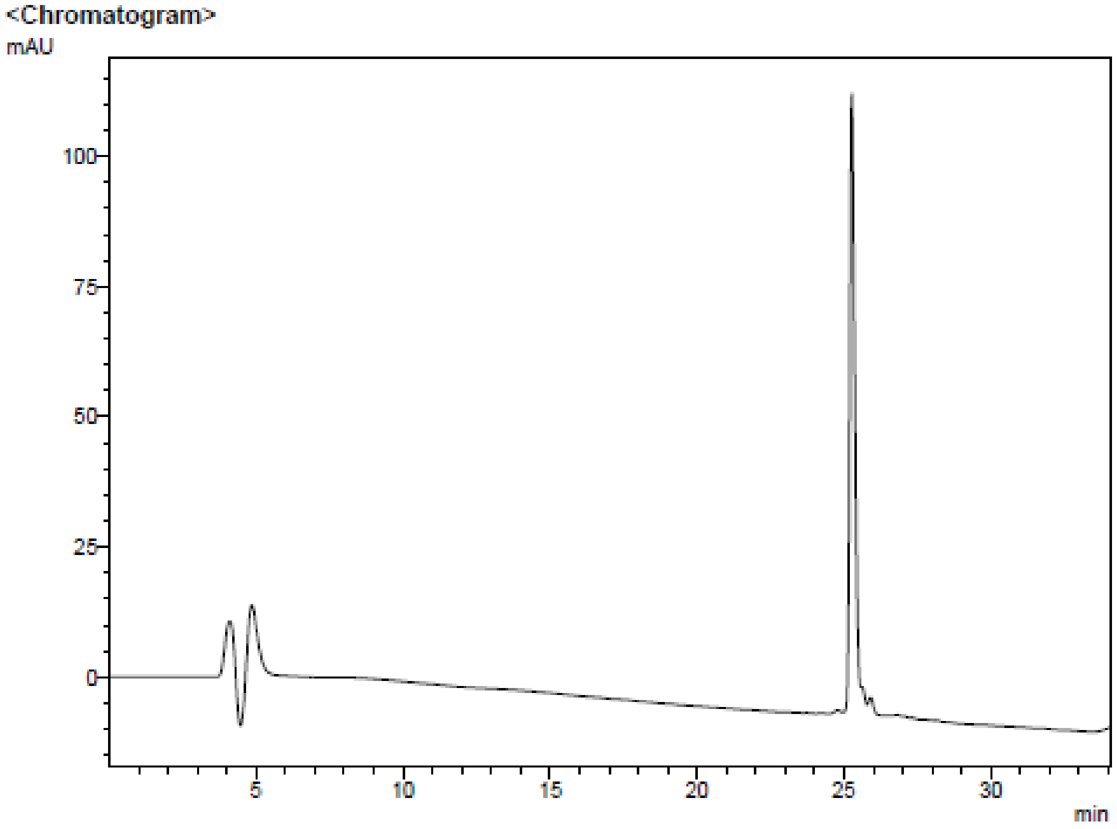

Figure S1 HPLC chromatogram of purified M_1 (The originally described major product of tridecaptin M). The peaks at 5 min in this chromatogram or other HPLC profiles belong to solvent.

Sample Name	: M152-P1 pure (XBridgeC18, 1.0 ml/min)
Sample ID	: 10-35%ACN/20%ACN0.075%TFA
Vail#	: 151
Injection Volume	: 30 uL
Data Filename	: Sample_322.lcd
Method Filename	: manoj.lcm
Batch Filename	:
Date Acquired	: 4/19/2019 12:52:39 PM
Data Processed	: 4/19/2019 2:27:21 PM

<Chromatogram>

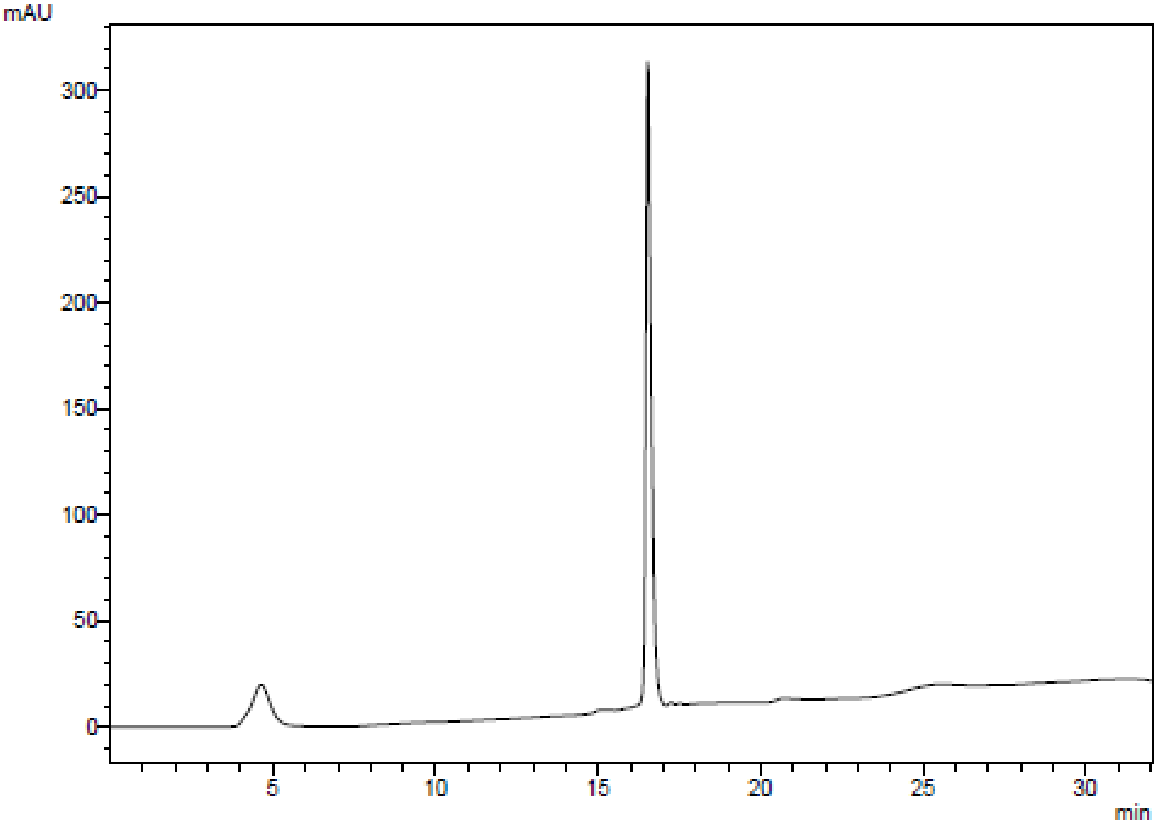

Figure S2 HPLC chromatogram of purified M_2

Sample Name	: M152-F3(1) pure (XBridgeC18, 1.0 ml/min)
Sample ID	: 10-35%ACN/20%ACN0.075%TFA
Vail#	: 151
Injection Volume	: 30 uL
Data Filename	: Sample_323.lcd
Method Filename	: manoj.lcm
Batch Filename	:
Date Acquired	: 4/19/2019 1:25:58 PM
Data Processed	: 4/19/2019 2:28:23 PM

<Chromatogram>

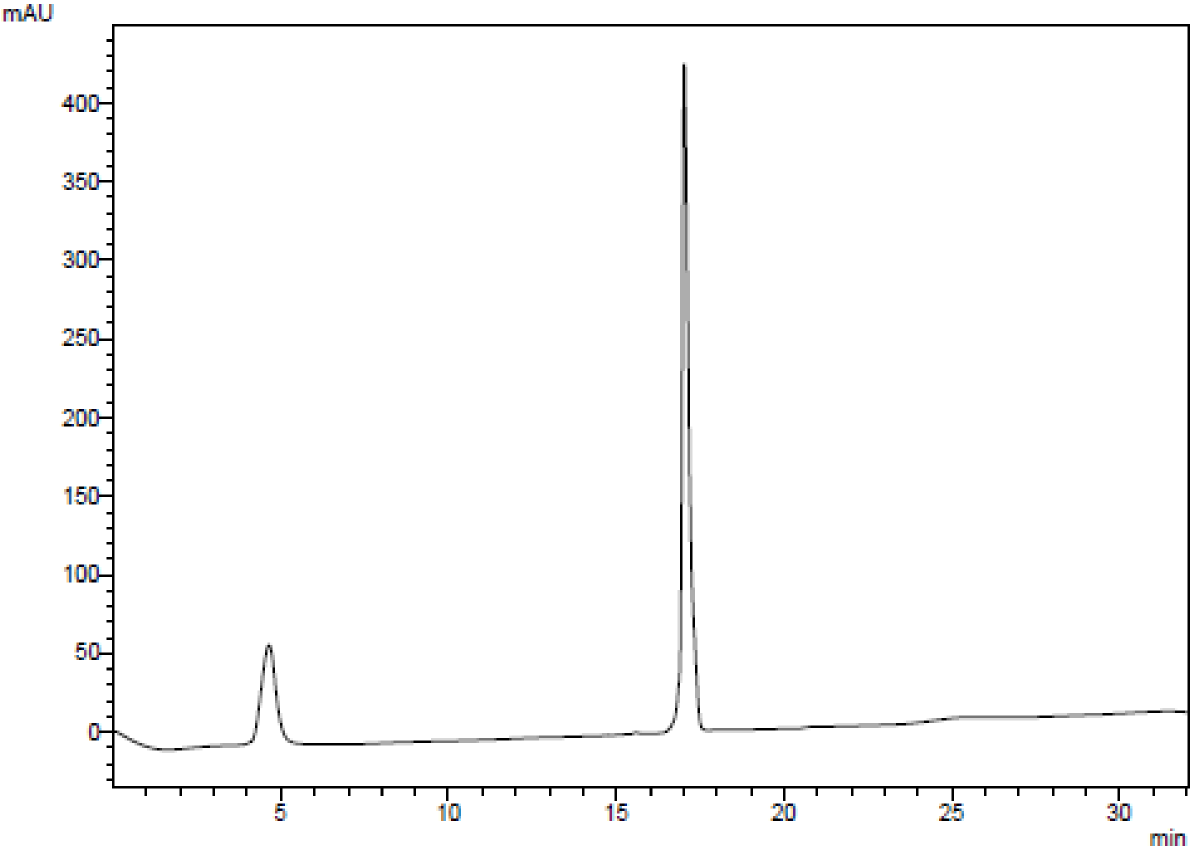

Figure S3 HPLC chromatogram of purified M_3

Sample Name	: M152-F3(2) pure (XBridgeC18, 1.0 ml/min)
Sample ID	: 10-35%ACN/20%ACN0.075%TFA
Vail#	: 151
Injection Volume	: 30 uL
Data Filename	: Sample_324.lcd
Method Filename	: manoj.lcm
Batch Filename	:
Date Acquired	: 4/19/2019 2:08:58 PM
Data Processed	: 4/19/2019 2:41:03 PM

<Chromatogram>

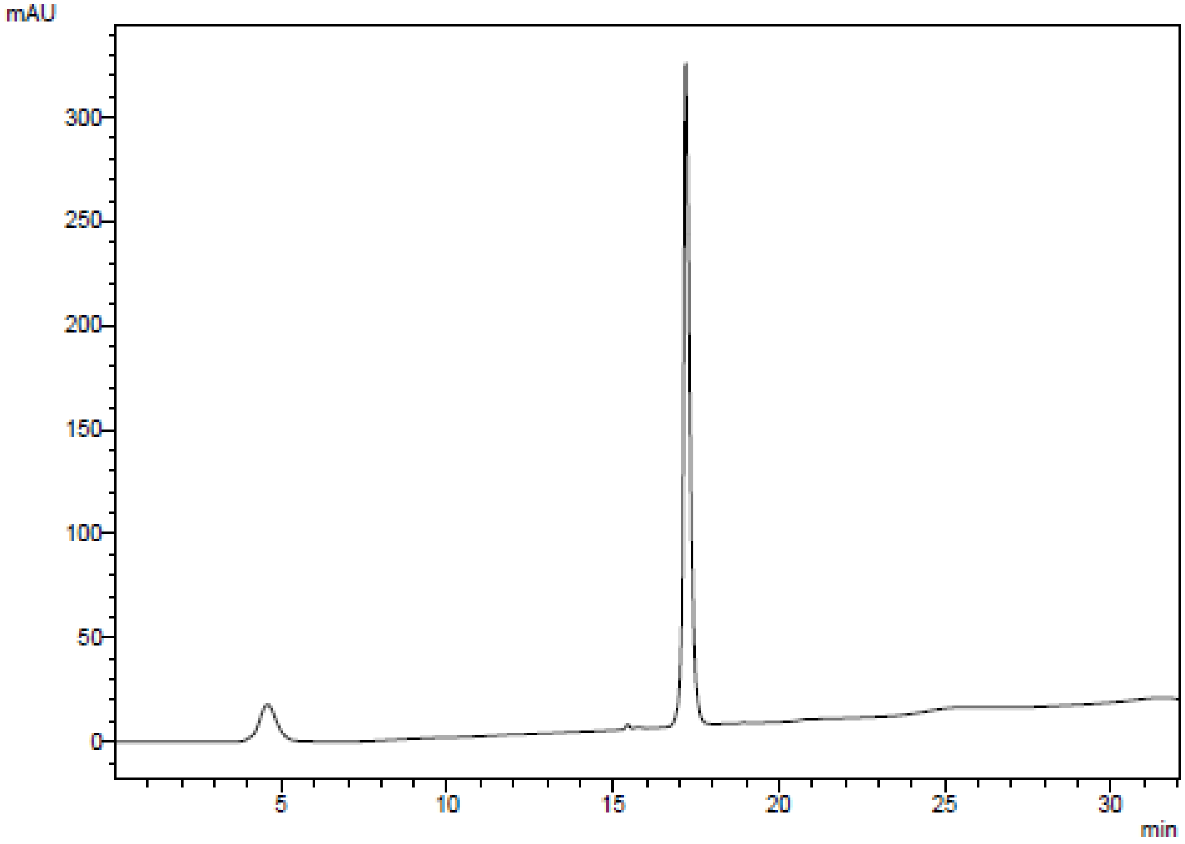

Figure S4 HPLC chromatogram of purified M_4

Sample Name	: M152-F4 pure (XBridgeC18, 1.0 ml/min)
Sample ID	: 10-35%ACN/20%ACN0.075%TFA
Vail#	: 151
Injection Volume	: 30 uL
Data Filename	: Sample_325.lcd
Method Filename	: manoj.lcm
Batch Filename	:
Date Acquired	: 4/19/2019 2:47:55 PM
Data Processed	: 4/19/2019 3:20:00 PM

<Chromatogram>

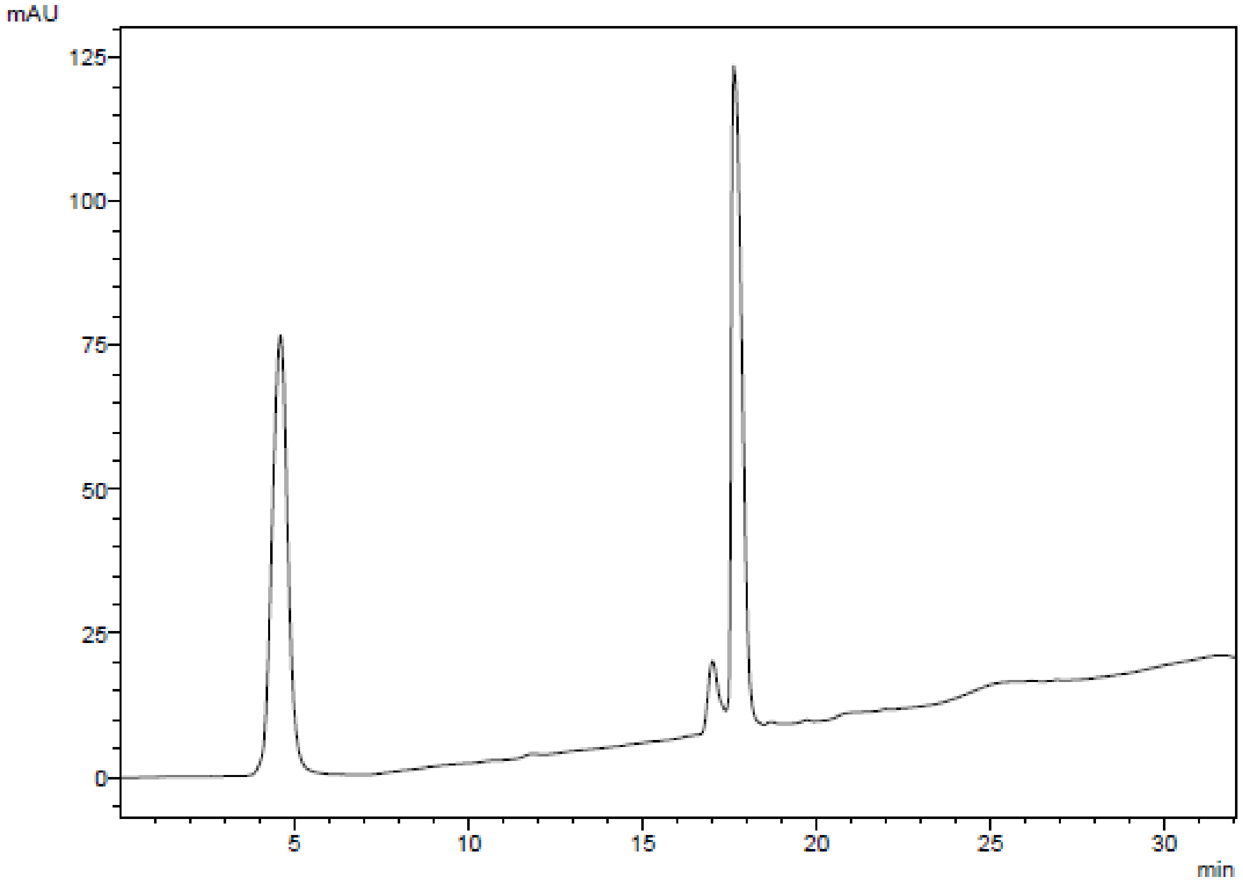

Figure S5 HPLC chromatogram of purified M_5

Sample Name	: P5-2 M152-P3 (Phenomenex C4 1.0ml/min)
Sample ID	: 3-35%ACN/Water 0.075%TFA
Vail#	: 153
Injection Volume	: 100 uL
Data Filename	: Sample_195.lcd
Method Filename	: manoj.lcm
Batch Filename	:
Date Acquired	: 3/30/2019 5:48:03 PM
Data Processed	: 3/30/2019 7:38:04 PM

<Chromatogram>

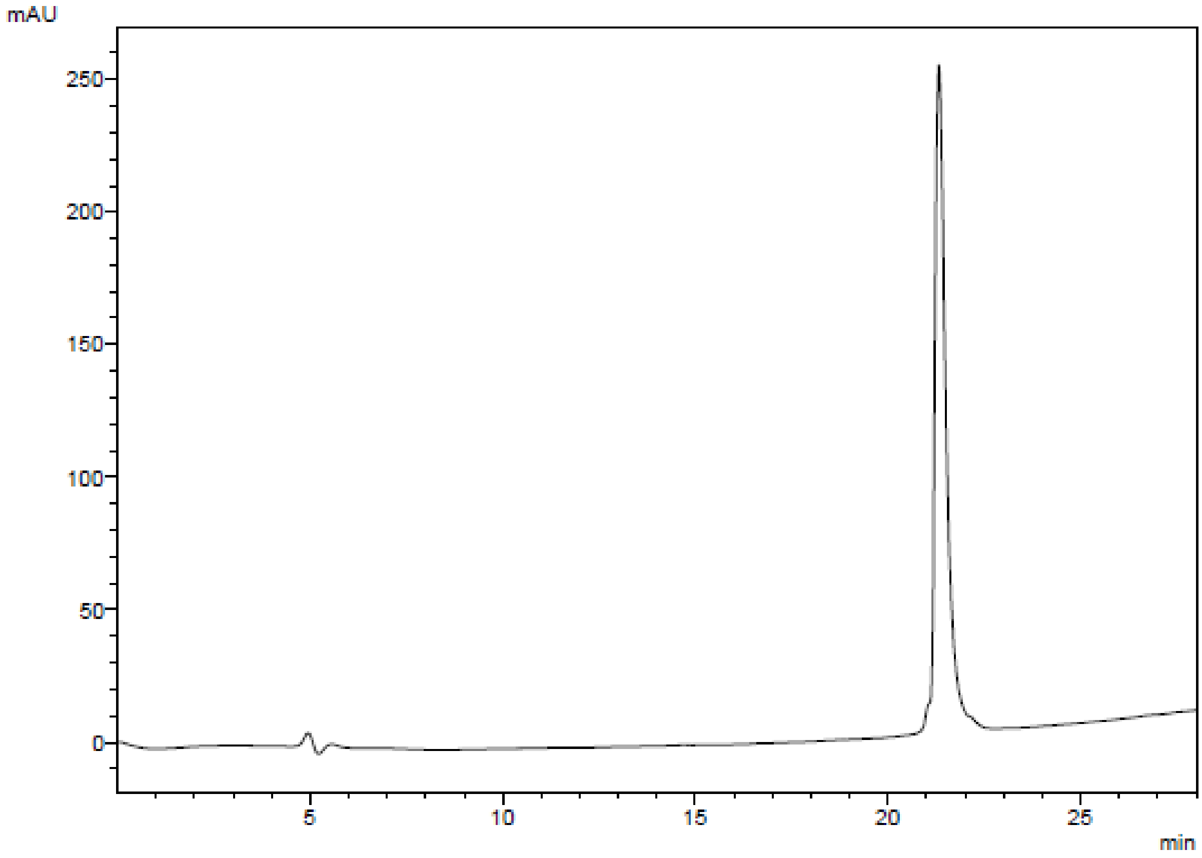

Figure S6 HPLC chromatogram of purified M_6

Sample Name	: M152-P6 pure (Xbridge C18 1.0ml/min)
Sample ID	: 10-28%ACN/water 0.075%TFA
Vail#	: 151
Injection Volume	: 30 uL
Data Filename	: Sample_233.lcd
Method Filename	: manoj.lcm
Batch Filename	:
Date Acquired	: 4/3/2019 7:22:12 PM
Data Processed	: 4/10/2019 7:29:43 PM

<Chromatogram>

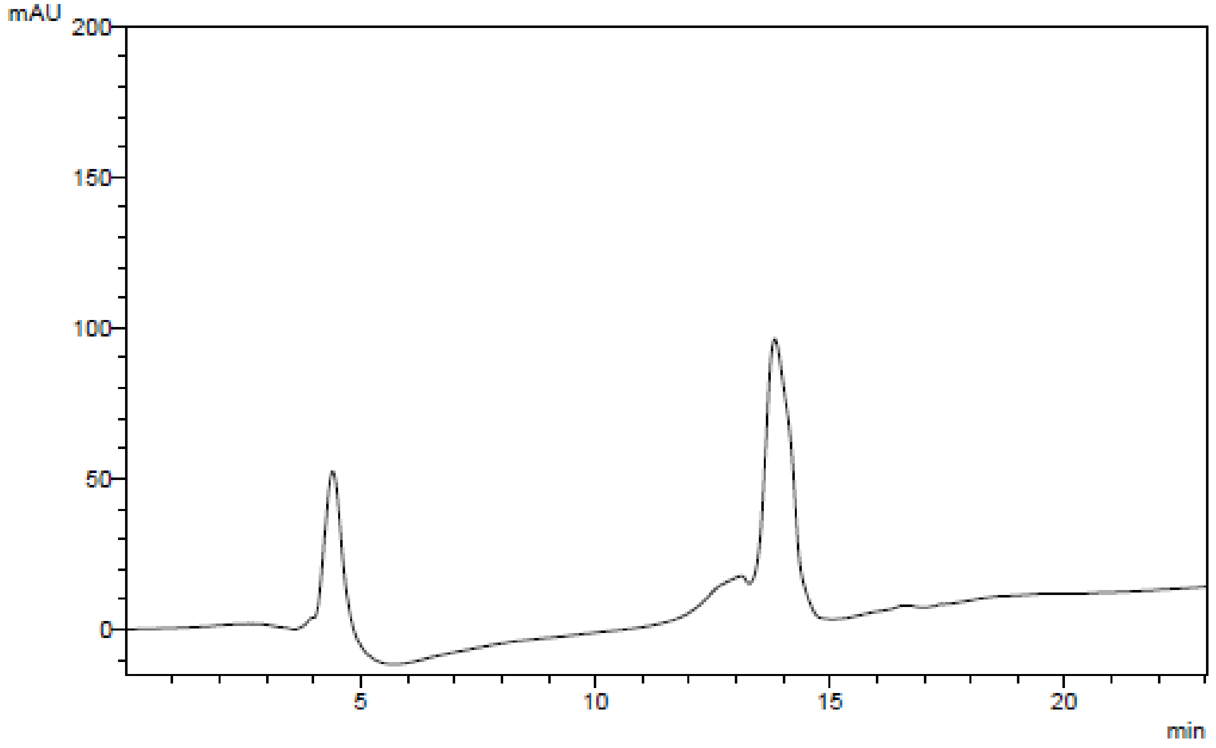

Figure S7 HPLC chromatogram of purified M_7

Sample Name	: P7
Sample ID	: Unknown conc
Vail#	: 152
Injection Volume	: 30 uL
Data Filename	: Sample_116.lcd
Method Filename	: manoj.lcm
Batch Filename	: Manoj Q.lcb
Date Acquired	: 3/15/2019 9:24:43 PM
Data Processed	: 10/18/2019 3:28:18 PM

<Chromatogram>

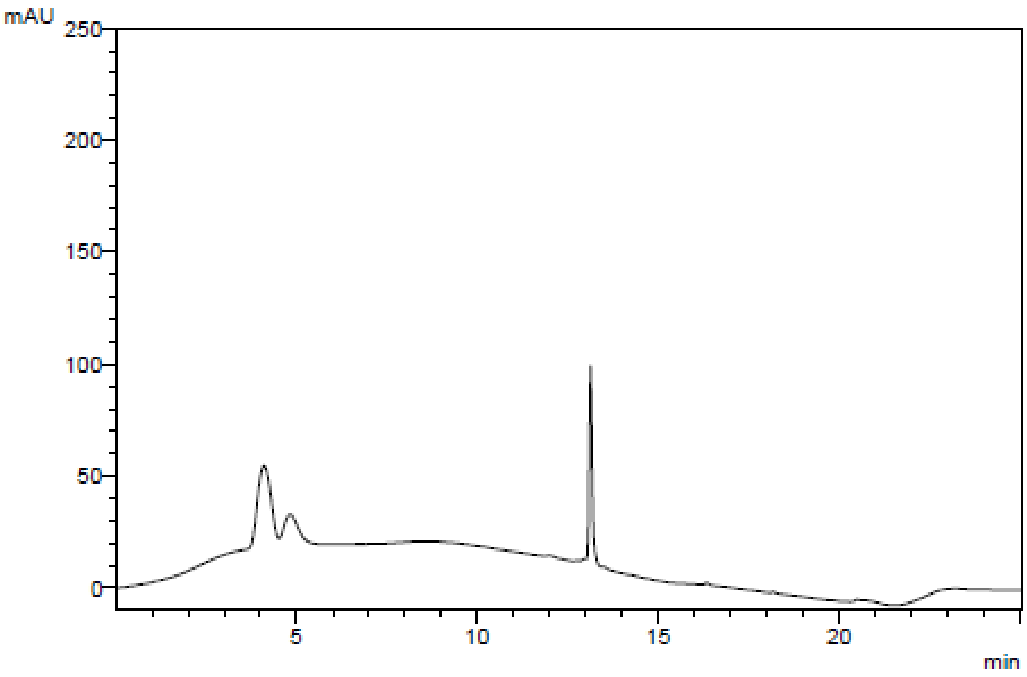

Figure S8 HPLC chromatogram of purified M_8

Sample Name	: M152-P10 pure (XBridgeC18, 1.0 ml/min)
Sample ID	: 10-35%ACN/20%ACN0.075%TFA
Vail#	: 151
Injection Volume	: 30 uL
Data Filename	: Sample_326.lcd
Method Filename	: manoj.lcm
Batch Filename	:
Date Acquired	: 4/19/2019 3:30:34 PM
Data Processed	: 4/19/2019 4:08:06 PM

<Chromatogram>

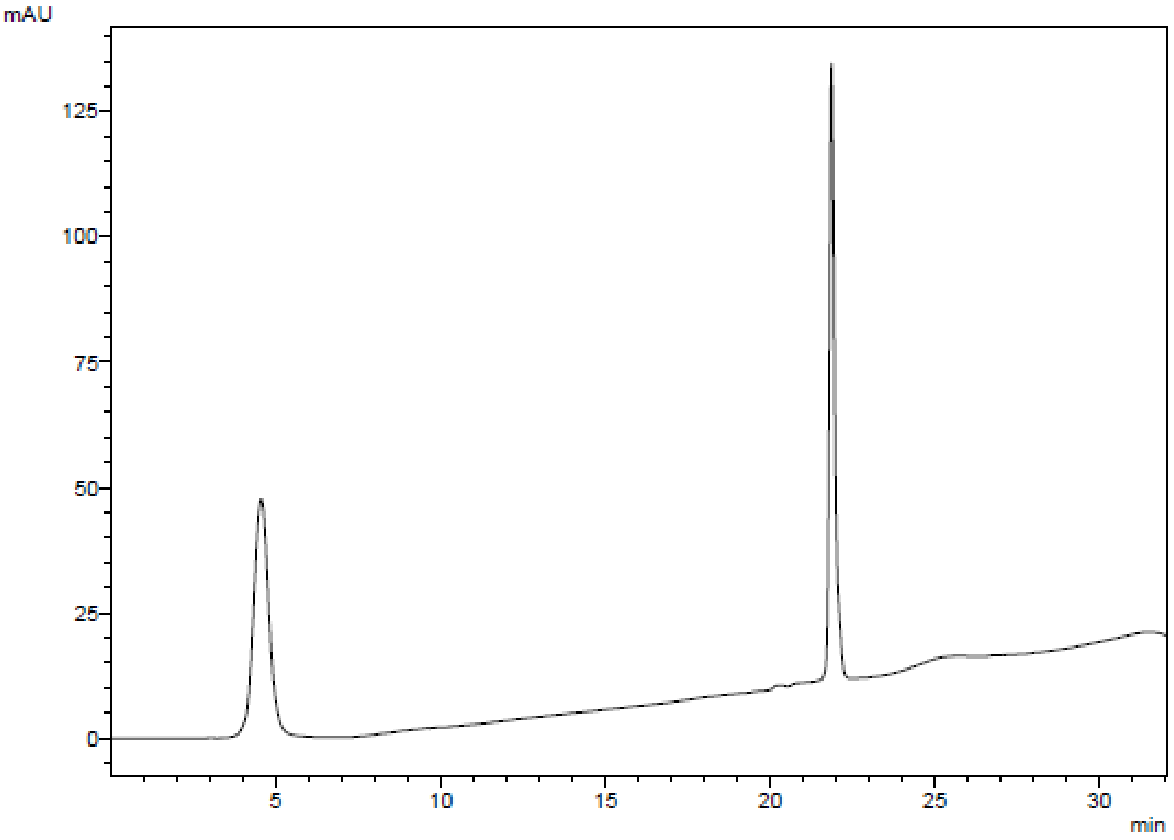

Figure S9 HPLC chromatogram of purified M_{10}

Sample Name : M152-P11 pure (XBridgeC18, 1.0 ml/min)
Sample ID : 10-35%ACN/20%ACN0.075%TFA
Vail# : 151
Injection Volume : 30 uL
Data Filename : Sample_388.lcd
Method Filename : manoj.lcm
Batch Filename :
Date Acquired : 4/22/2019 8:33:13 PM
Data Processed : 4/29/2019 1:40:47 PM

<Chromatogram>

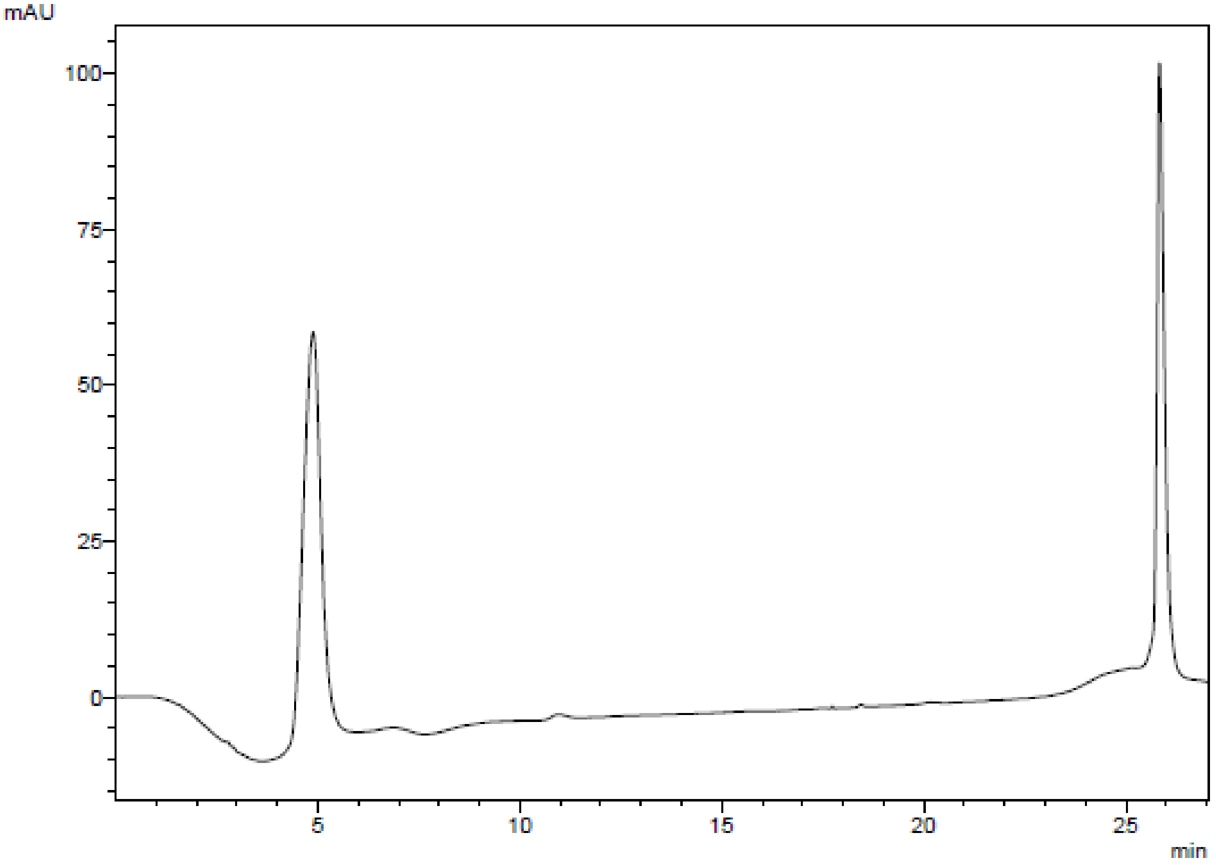

Figure S10 HPLC chromatogram of purified M_{11}

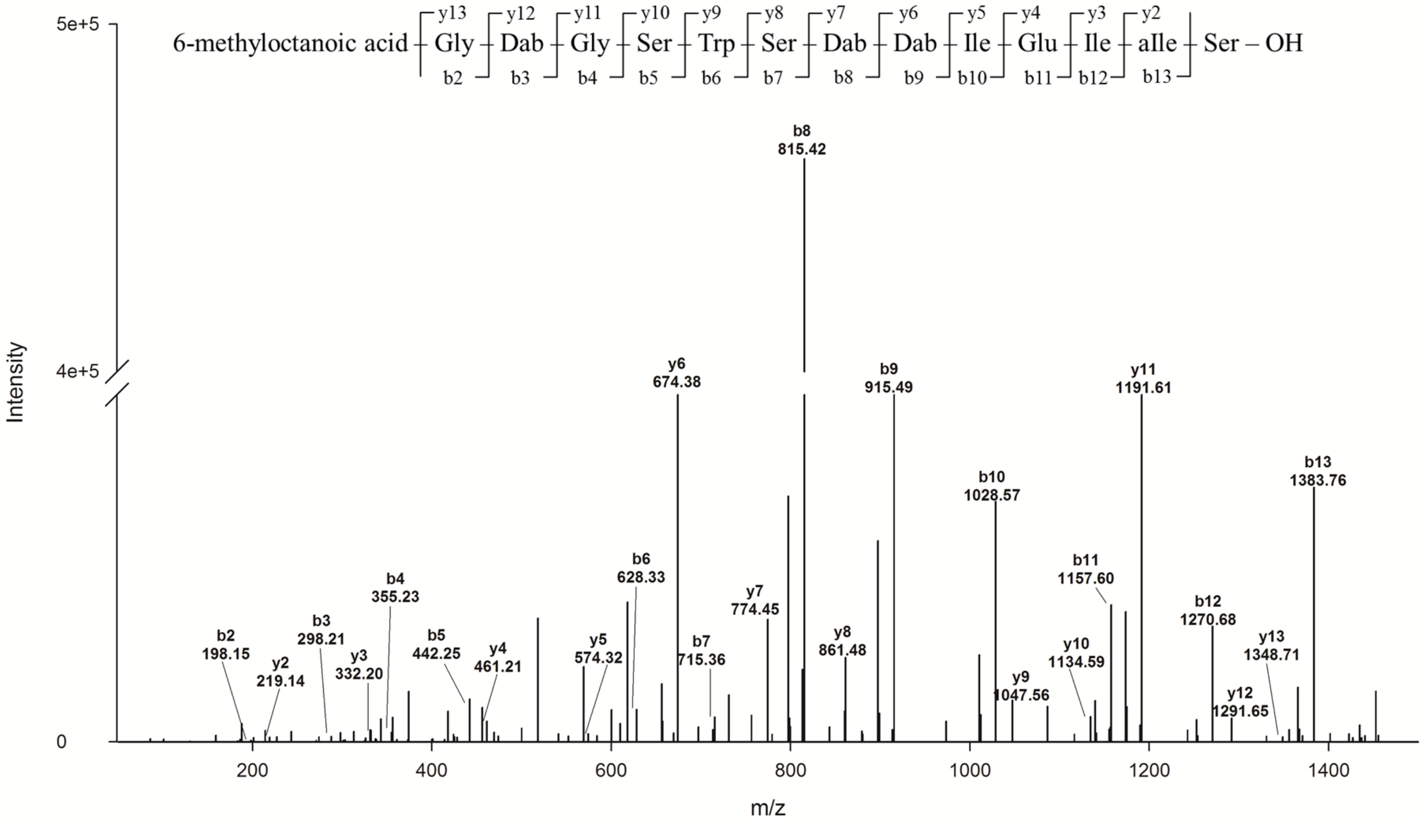

Figure S11 Tandem MS spectroscopic data of tridecaptin M (or M_1). This data is already published by our group in original research paper on tridecaptin M[1]. The MS/MS data of rest of the variants was analyzed relative to this spectrum.

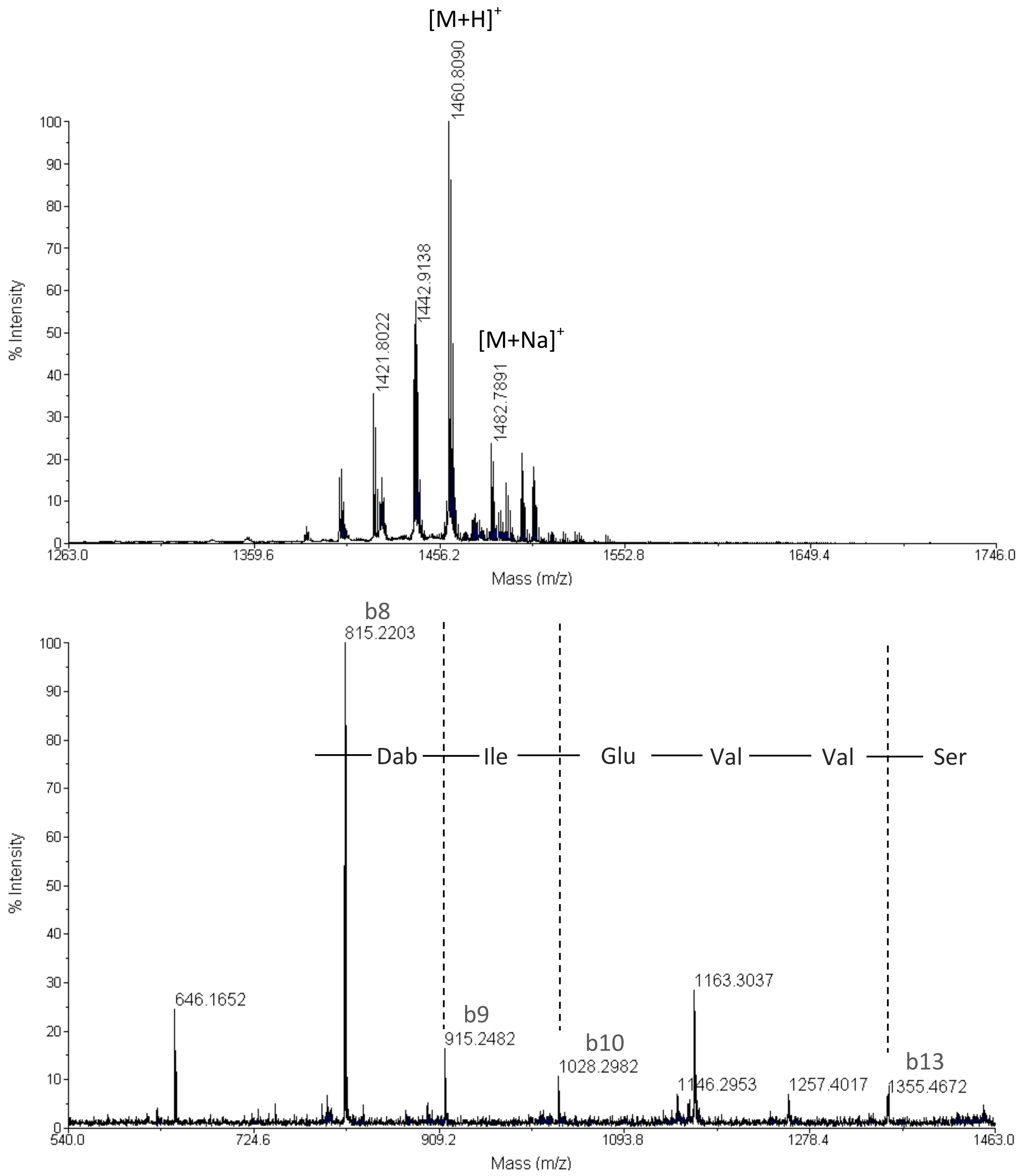

Figure S12 Mass spectroscopic data of M_2. Top, MS data; bottom, MS/MS spectrum

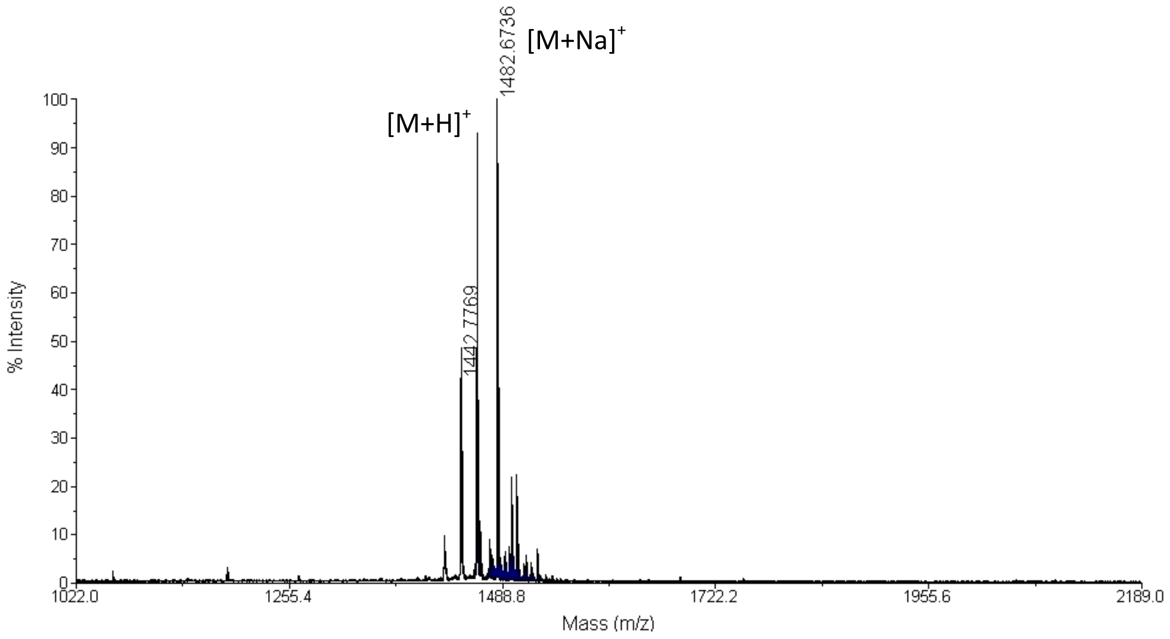

Figure S13 Mass spectroscopic data of M_3

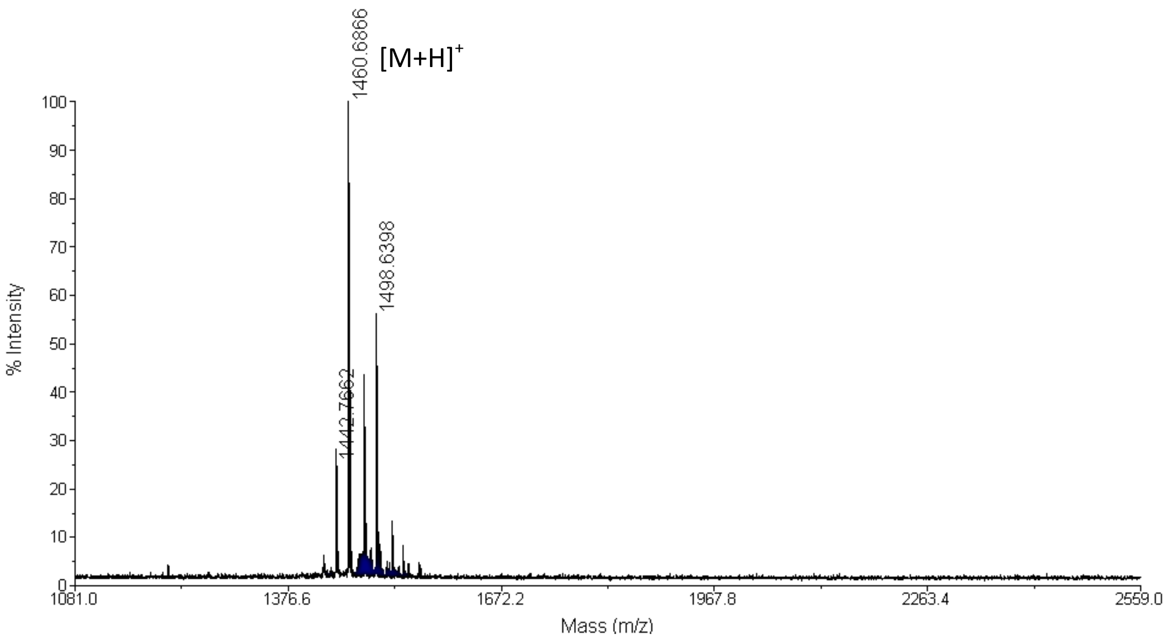

Figure S14 Mass spectroscopic data of M_4

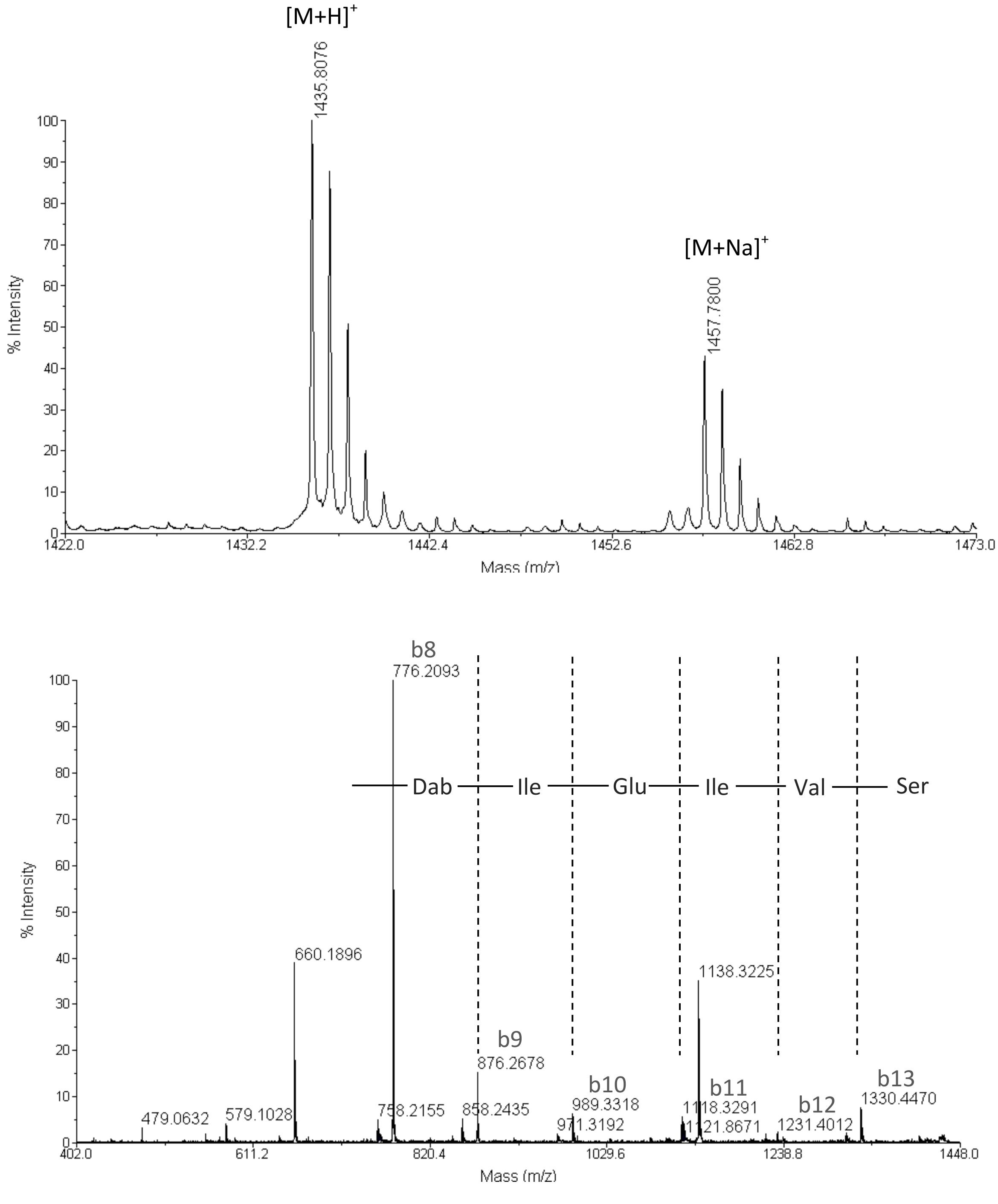

Figure S15 Mass spectroscopic data of M_5. Top, MS data; bottom, MS/MS spectrum

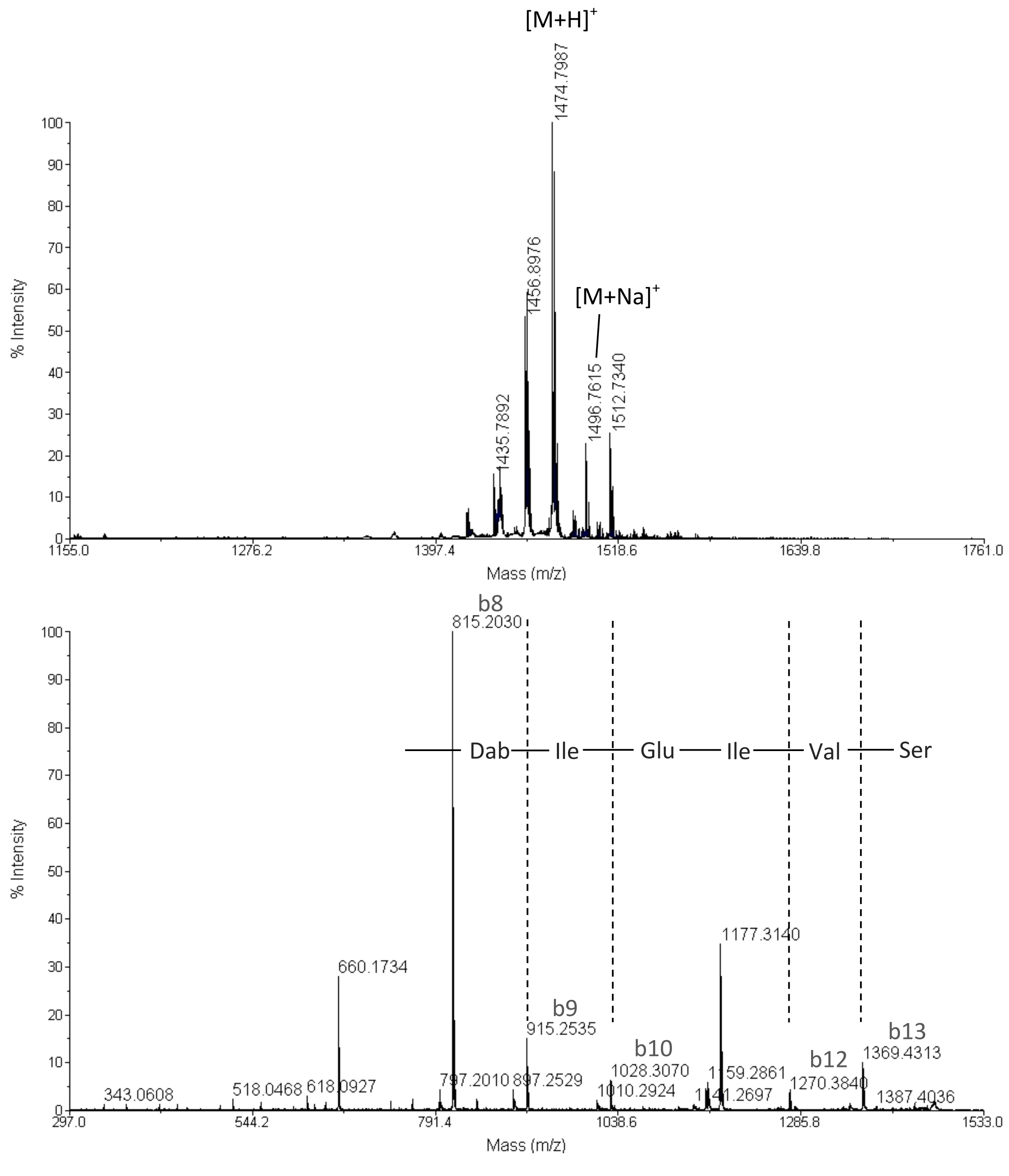

Figure S16 Mass spectroscopic data of M_6. Top, MS data; bottom, MS/MS spectrum

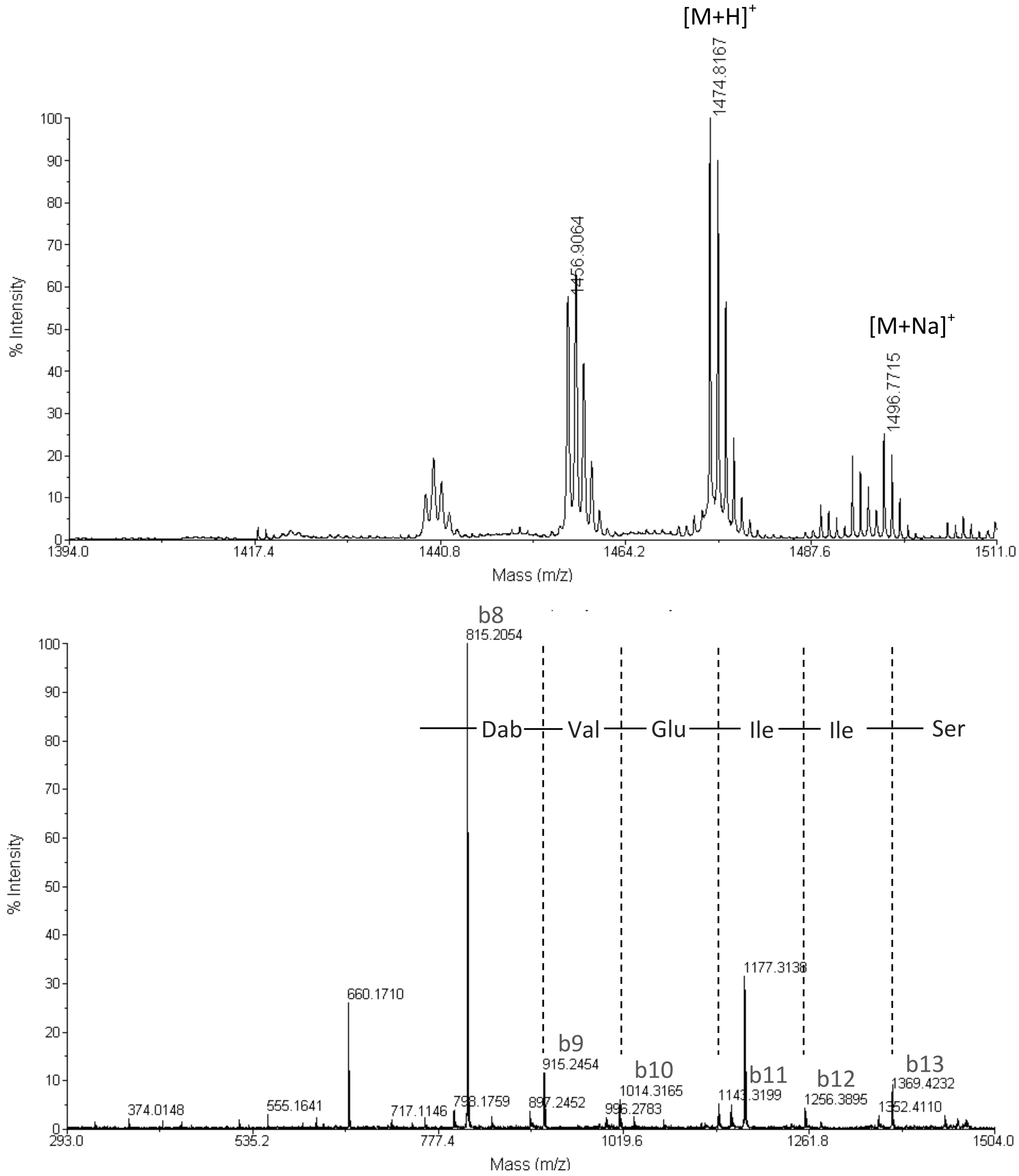

Figure S17 Mass spectroscopic data of M_7. Top, MS data; bottom, MS/MS spectrum

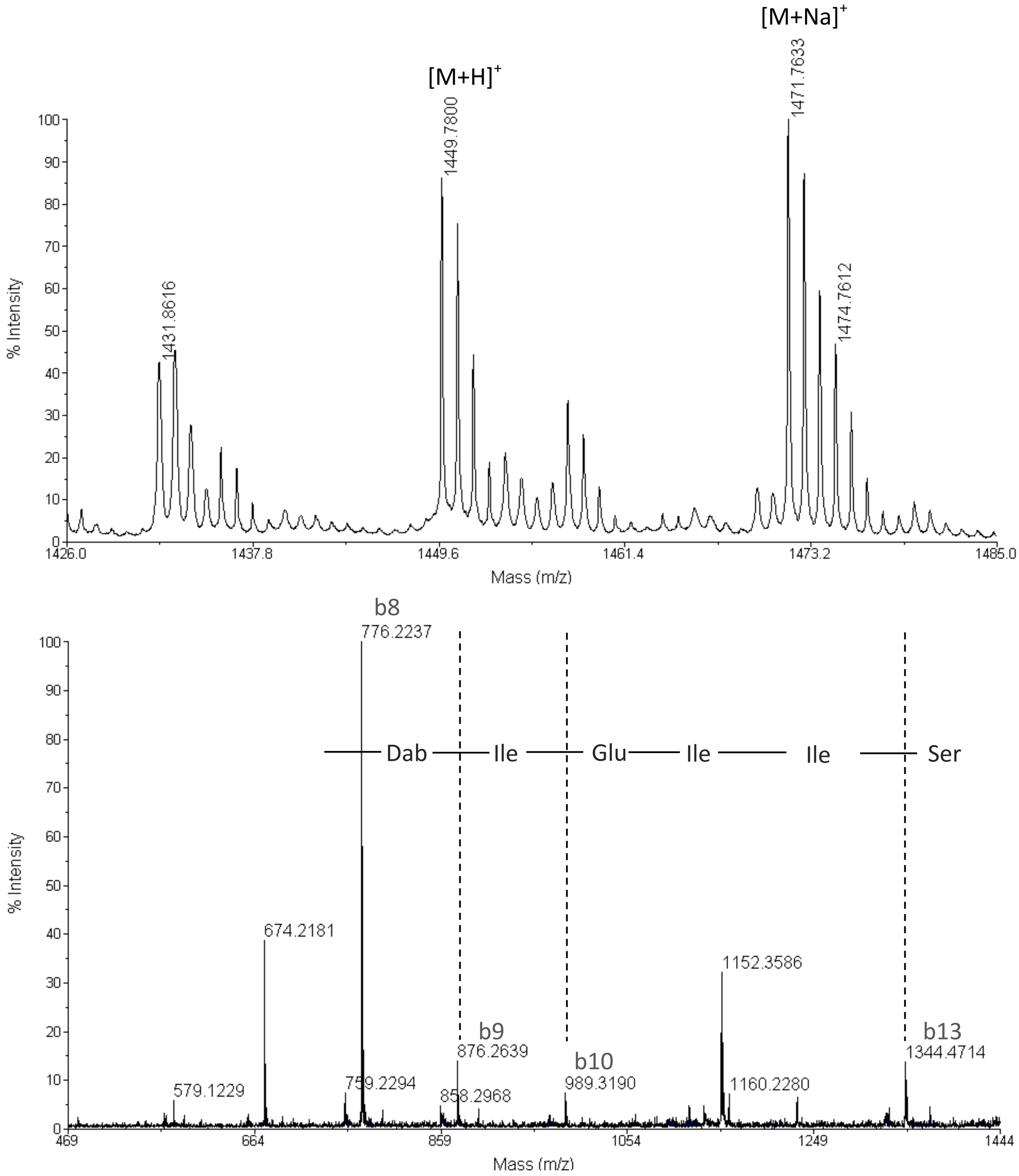

Figure S18 Mass spectroscopic data of M_8. Top, MS data; bottom, MS/MS spectrum

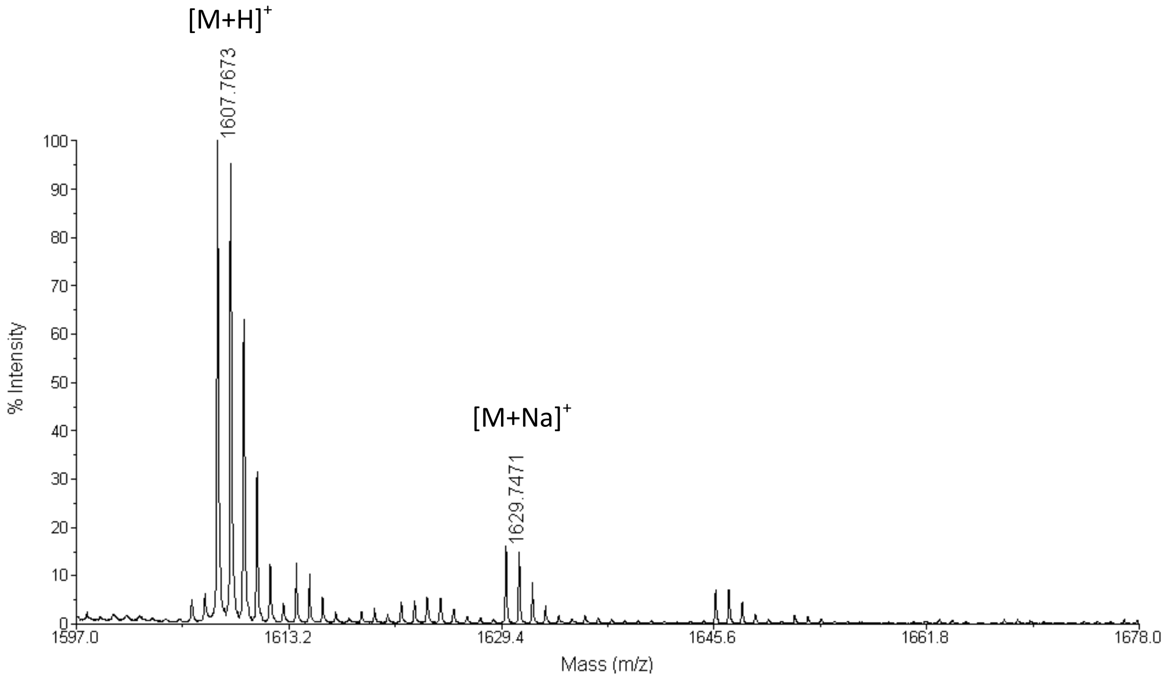

Figure S19 Mass spectroscopic data of M_9

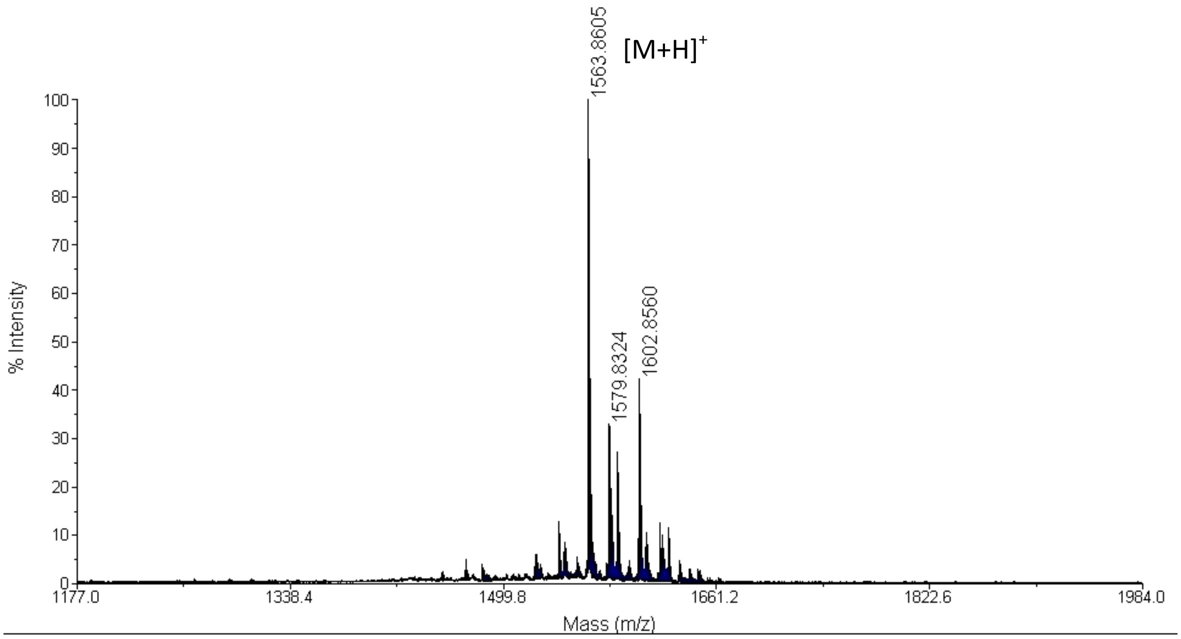

Figure S20 Mass spectroscopic data of M_{10}

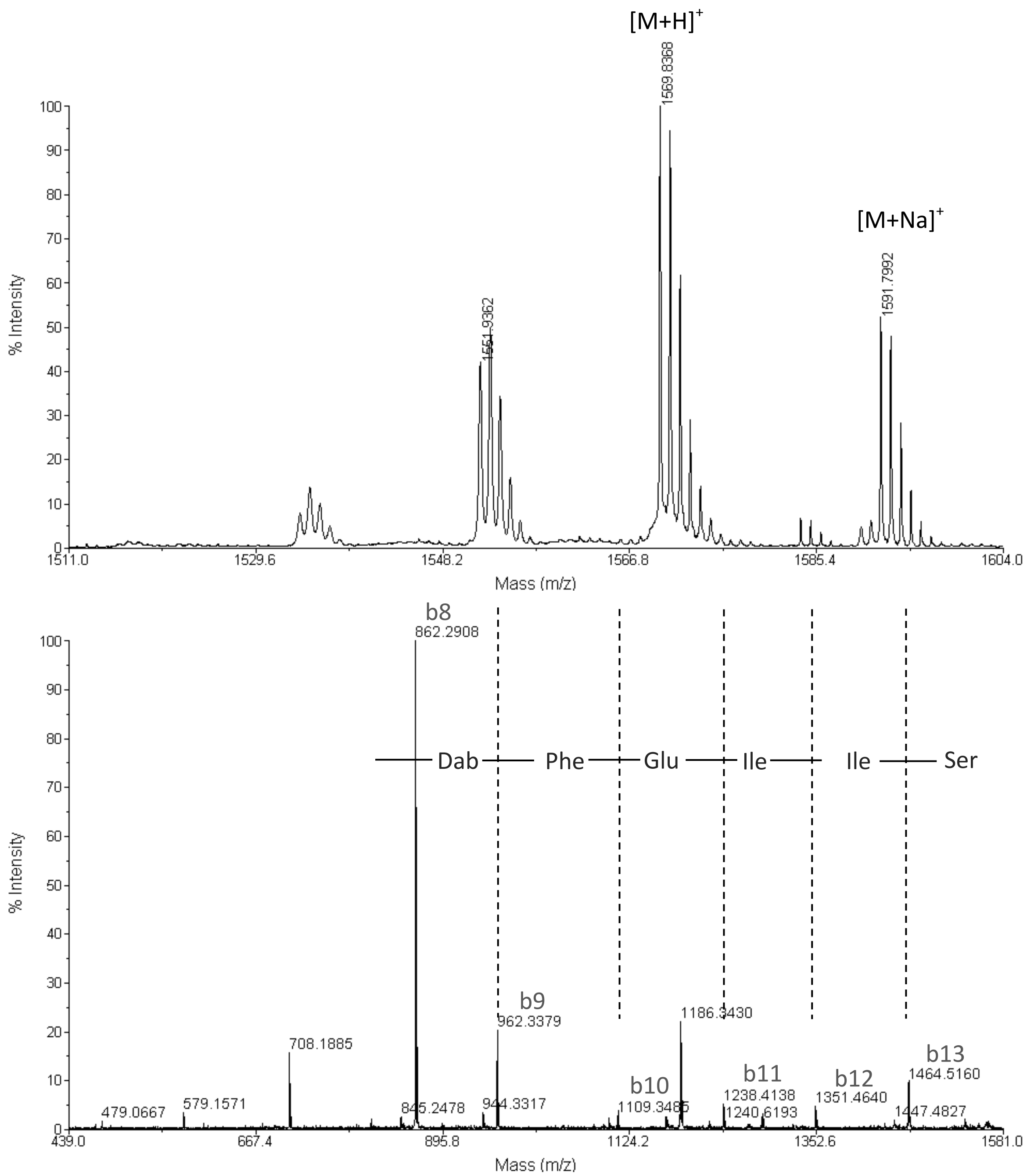

Figure S21 Mass spectroscopic data of M_{11}. Top, MS data; bottom, MS/MS spectrum

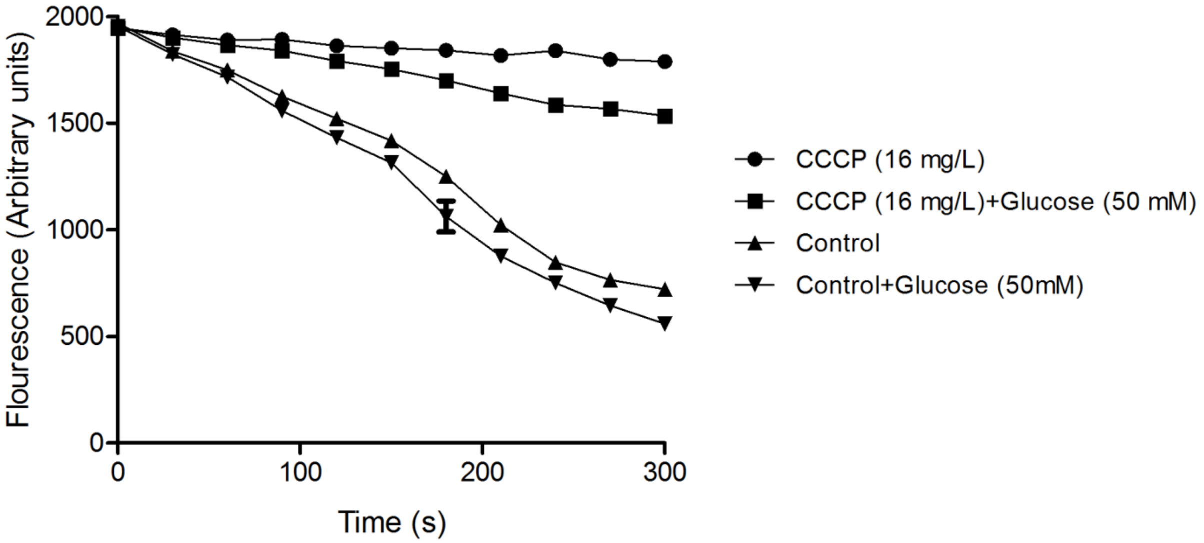

Figure S22 Nile red efflux inhibition assay in *A. baumannii* ATCC 19606 to determine the presence of efflux pumps. *A. baumannii* was incubated with Nile red dye in the presence or absence of efflux pump inhibitor, CCCP, and the fluorescence of the intracellular dye was measured. Treated cells showed inhibition of efflux which indicates the presence of efflux pumps in the bacteria. Glucose re-energizes the efflux pumps and thereby reducing the activity of CCCP.

References

1 Jangra, M. *et al.* Tridecaptin M, a new variant discovered in mud bacterium, shows activity against colistin-and extremely drug-resistant Enterobacteriaceae. *Antimicrob. Agents Chemother.* **63**, e00338-00319 (2019).
